# Hypoxia-induced epigenetic regulation of miR-485-3p promotes stemness and chemoresistance in pancreatic ductal adenocarcinoma via SLC7A11-mediated ferroptosis

**DOI:** 10.1038/s41420-024-02035-x

**Published:** 2024-05-29

**Authors:** Xinxin Liu, Zhihua Huang, Qiuzheng Chen, Kai Chen, Weikang Liu, Guangnian Liu, Xiangyu Chu, Dongqi Li, Yongsu Ma, Xiaodong Tian, Yinmo Yang

**Affiliations:** 1 Department of General Surgery, Peking University First Hospital, Beijing, 100034 China; 2 Department of Biochemistry and Molecular Biology, School of Basic Medical Sciences, Peking University, Beijing, 100191 China

**Keywords:** Pancreatic cancer, Gene regulation

## Abstract

The mechanism of hypoxia in chemoresistance of pancreatic ductal adenocarcinoma (PDAC) remains elusive. In this study, we revealed the essential role of miR-485-3p in PDAC, particularly its impact on cancer stemness and gemcitabine resistance under hypoxic conditions. We found substantial downregulation of miR-485-3p in PDAC tissues, with lower expression correlating to poor patient outcomes. Mechanistically, miR-485-3p influenced stemness characteristics, as evidenced by reduced tumor-sphere formation and increased sensitivity to gemcitabine upon overexpression. Moreover, we identified SOX9 and SLC7A11 as two targets of miR-485-3p, which play a vital role in stemness and ferroptosis. Under the hypoxic condition, DNMT3B expression was upregulated, leading to hypermethylation of the miR-485-3p promoter region. The reduced miR-485-3p expression promoted stemness and chemoresistance of PDAC. In conclusion, our findings elucidate the intricate interplay of hypoxia, epigenetic modifications, and ferroptosis in PDAC and shed light on potential avenues for targeted interventions that modulate cancer stemness and chemosensitivity, offering prospects for improved therapeutic strategies for PDAC.

## Introduction

Pancreatic ductal adenocarcinoma (PDAC) is a highly aggressive gastrointestinal malignancy with a dismal prognosis [1]. PDAC is currently the 4th leading cause of cancer-related death, and a 5-year survival rate is only approximately 11% [2]. Gemcitabine is still the cornerstone of chemotherapeutic agent for PDAC [3]. However, the response to chemotherapy agents is hampered by the drug resistance of PDAC. Hypoxia is a common occurrence in PDAC due to poor vascularization, uncontrolled growth, and fibrotic stroma [3]. Accumulating evidence has revealed that hypoxia is associated with stemness maintenance, gemcitabine resistance, and pathological angiogenesis [4, 5]. Our recent study demonstrated that hypoxic conditions in PDAC promote tumor angiogenesis by suppressing GJA1 expression [6]. However, molecular mechanisms modulating PDAC cell’s chemoresistance in hypoxic conditions remain unclear.

Recent studies have underscored the important role of microRNAs (miRNAs) in regulating proliferation, migration, invasion, stemness, and chemotherapy resistance in various tumors, including pancreatic cancer [7–10]. MiRNAs are a class of endogenous small non-coding RNAs typically composed of 19-24 nucleotides and exert regulatory effects by inhibiting mRNA translation or inducing mRNA degradation [11]. Moreover, hypoxic environments can affect the expression of miRNAs [12, 13]. MiR-485-3p, derived from the MIR-485 gene located on chromosome 14q32.31, has been implicated in breast cancer, osteosarcoma, prostate cancer, gastric cancer, hepatocellular carcinoma, and other tumors due to its aberrant expression [14–16]. In our previous study, aberrant expression of miR-485-3p inhibited the migration and invasion of PDAC cells [17], but the mechanism underlying its aberrant expression remains unclear.

In this study, we found that SOX9 and SLC7A11 are the downstream targets of miR-485-3p and DNMT3B downregulates miR-485-3p expression. DNMT3B/miR-485-3p/SLC7A11 axis promotes stemness maintenance and induces gemcitabine resistance in PDAC cells under hypoxic conditions.

## Results

### miR-485-3p is inhibited in PDAC cell lines under hypoxia conditions and associated with good clinical prognosis

**Fig. 1:**
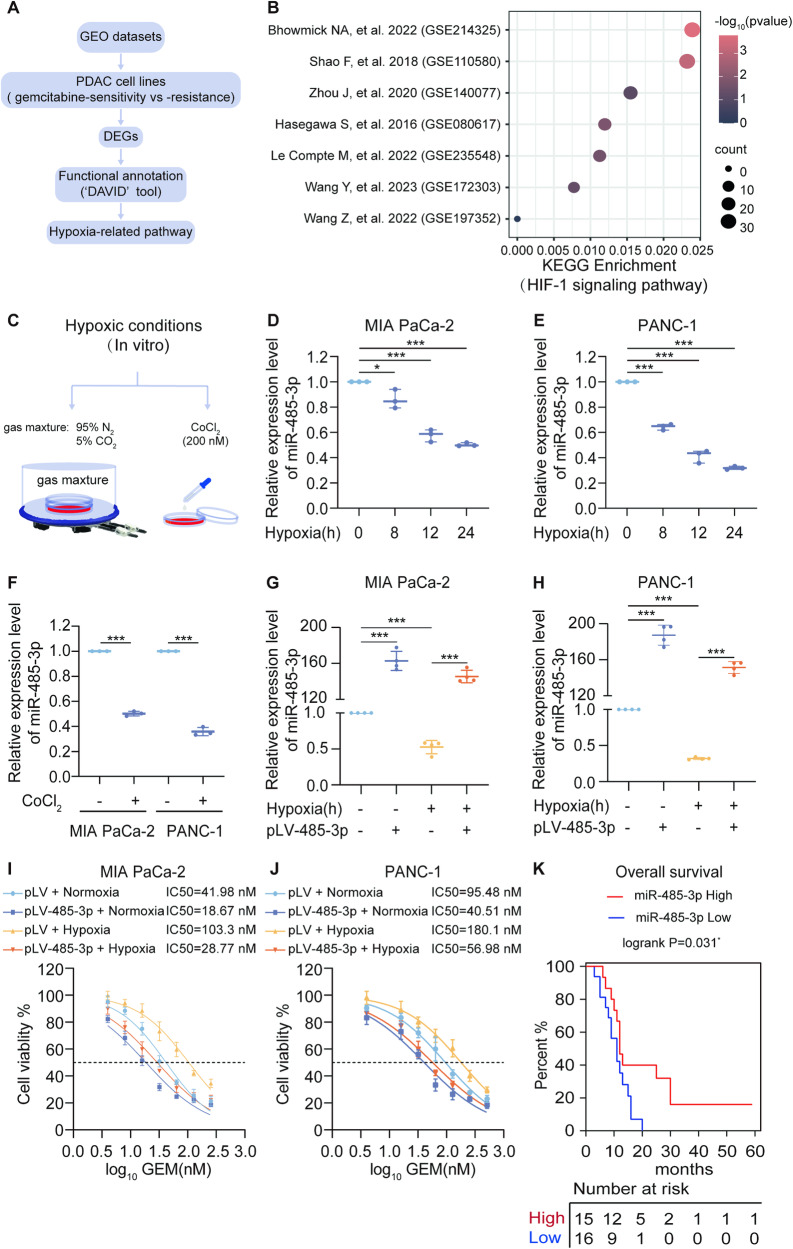
miR-485-3p is associated with good clinical prognosis and downregulated in PDAC cells under hypoxia. **A** Schematic of the screening strategy to identify the differential expression genes (DEGs) of gemcitabine -sensitive and -resistant pancreatic cancer cells in GEO datasets. **B** Bubble Chart was shown to visualize the KEGG enrichment of DEGs. **C** Hypoxic chamber or 200 µM CoCl_2_ method was used to induce hypoxic conditions for PDAC cells in vitro.**D**–**H** qPCR was used to detect miR-485-3p expression in MIA PaCa-2 and PANC-1 cells. **I**, **J** Gemcitabine dose-response curve showed the effect of miR-485-3p overexpression on the chemosensitivity of Mia Paca-2 (**I**) and PANC-1 cells (**J**) under normoxic or hypoxic conditions. **K** Kaplan–Meier survival curve analysis of miR-485-3p expression and the prognosis of PDAC patients. Data are expressed as mean ± SD from three or more than three independent experiments. *P < 0.05; **P < 0.01; ***P < 0.001.

To evaluate whether hypoxia-related signaling pathways are involved in gemcitabine resistance in pancreatic cancer cells, we used the GEO database to screen the differential expression genes (DEGs) of gemcitabine-sensitive and -resistant pancreatic cancer cells and the workflow was presented in Fig. 1A. The results showed that these DEGs had a correlation with HIF signaling pathway in most datasets, except for the GSE197352 dataset (Fig. 1 B). Next, we employed CoCl_2_ induced- or physical- method to imitate hypoxic condition in vitro (Fig. 1C). While HIF-1α was upregulated under hypoxia (Supplementary Fig. 1A, B), we found a significant downregulation of miR-485-3p expression (Fig. 1D–F).

We evaluated the impact of miR-485-3p on the chemosensitivity of PDAC cells. The results showed that miR-485-3p overexpression under normoxia or hypoxia (Fig. 1G, H), increased the sensitivity of PDAC cells to gemcitabine and partially reversed hypoxia-induced resistance of PDAC to gemcitabine (Fig. 1I, J). Moreover, we established drug-resistant cell lines and observed lower miR-485-3p expression in these cells (Fig. S2A, C). Overexpression of miR-485-3p in drug-resistant cell lines reversed the resistance of PDAC cells to gemcitabine (Fig. S2B, D). Furthermore, lower miR-485-3p expression was closely correlated with reduced overall survival in PDAC (Fig. 1K). Collectively, these data indicated that miR-485-3p exhibited aberrant expression patterns in PDAC tissues and cells, and was downregulated under hypoxic conditions.

### Hypoxia upregulates SOX9 and SLC7A11 expression by downregulating the expression of miR-485-3p

**Fig. 2:**
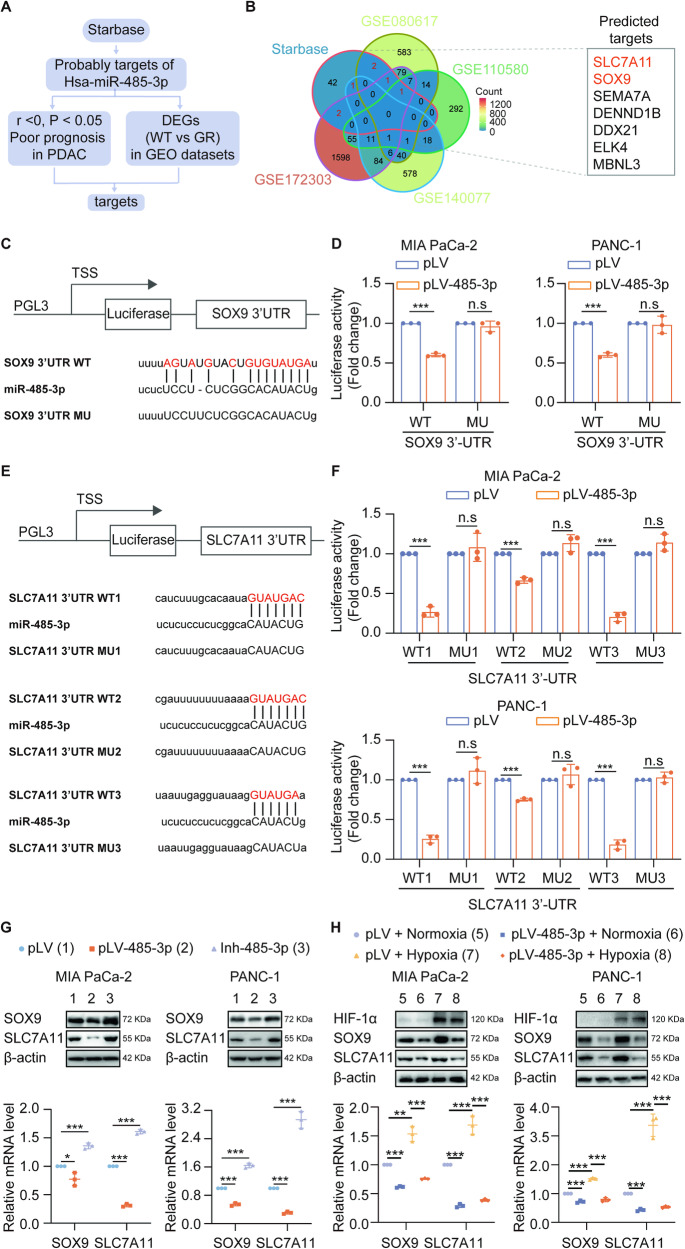
SOX9 and SLC7A11 are targets of miR-485-3p. **A** Schematic of the screening strategy to identify targets of miR-485-3p in Starbase source. **B** Venn diagram showing intersection genes (SOX9, SLC7A11) of wild-type and drug-resistant and predicted targets from Starbase. **C** The predicted binding sites of miR-485-3p in the 3’UTR of SOX9 and corresponding mutant sites of SOX9. **D** Relative luciferase activity was determined in Mia Paca-2 and PANC-1 cells transfected with wild-type or mutant 3′-UTR of SOX9. **E** Three predicted binding sites of miR-485-3p in the 3’UTR of SLC7A11 and corresponding mutant sites of SLC7A11. **F** Relative luciferase activity was determined in Mia Paca-2 and PANC-1 cells transfected with the wild-type or mutant 3′-UTR of SLC7A11. **G** miR-485-3p affected SOX9 and SLC7A11 mRNA and protein expression in Mia PaCa-2 and PANC-1 cells. **H** miR-485-3p overexpression affected SOX9 and SLC7A11 mRNA and protein expression in Mia PaCa-2 and PANC-1 cells under normoxia or hypoxia conditions. Data are expressed as mean ± SD from three independent experiments. *P < 0.05; **P < 0.01; ***P < 0.001.

To elucidate the molecular mechanism underlying the effects of miR-485-3p on gemcitabine resistance in PDAC cells under hypoxic conditions, we utilized the “Starbase” to predict potential targets of miR-485-3p (Fig. 2A and Supplementary Fig. 3A). Comparing the PDAC GEO datasets of DEGs between wild-type and drug-resistant cells, we found that SOX9 and SLC7A11were potential targets of miR-485-3p (Fig. 2B). Moreover, we analyzed the TCGA-PDAC dataset to confirm that both SOX9 and SLC7A11 expression was negatively correlated with miR-485-3p expression (Supplementary Fig. 3B, C). Luciferase assays further revealed that miR-485-3p overexpression inhibited the luciferase activity of the wild-type containing SOX9 (Fig. 2C, D). Moreover, there were three potential sites of miR-485-3p binding within the 3’-UTR region of SLC7A11 (Fig. 2E) and the luciferase activity of the binding site 3 was the lowest (Fig. 2F). MiR-485-3p overexpression inhibited the expression of SOX9 and SLC7A11 while silencing miR-485-3p had the opposite effects (Fig. 2G, Supplementary Fig 3D, E). Additionally, miR-485-3p could reverse hypoxia-induced SOX9 and SLC7A11 expression (Fig. 2H). Besides, we found that when miR-485-3p expression was silenced, the effect of oxygen concentration on the expression levels of SOX9 and SLC7A11 has no significant difference, indicating that the expression changes of SOX9 and SLC7A11 under hypoxia are mainly mediated by miR-485-3p (Supplement Fig. 4). Taken together, these results suggested that miR-485-3p could target SOX9 and SLC7A11 under hypoxic condition.

### miR-485-3p suppresses stemness of PDAC cells under hypoxic condition

**Fig. 3:**
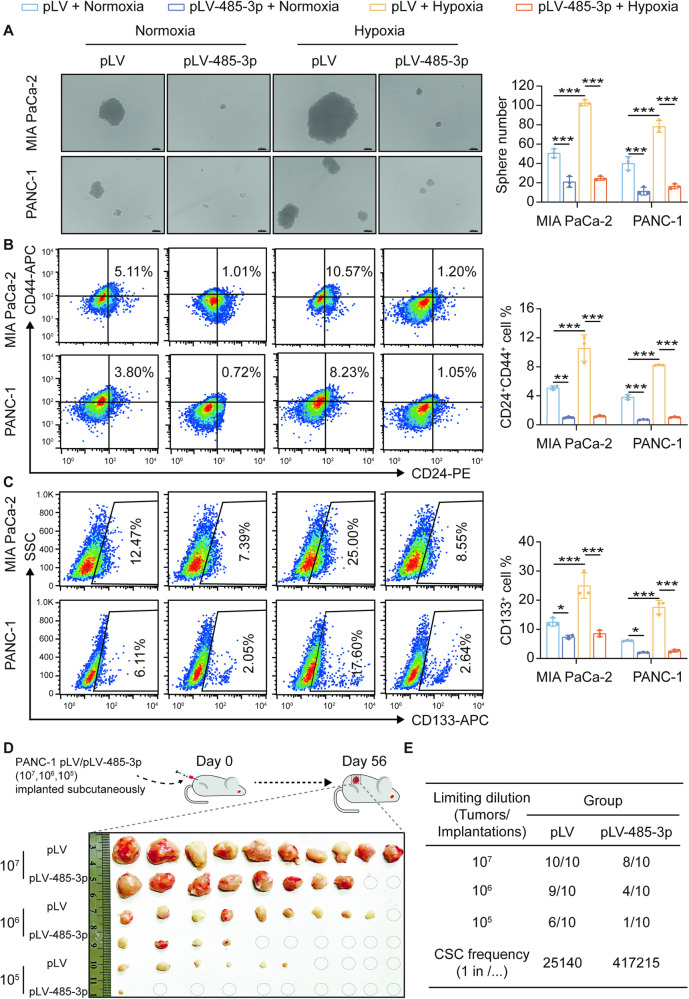
MiR-485-3p suppresses the stemness of PDAC cells under hypoxic conditions. **A** Effects of miR-485-3p overexpression on the tumor-sphere formation of PDAC cells MIA PaCa-2 and PANC-1 under normoxic or hypoxic conditions. Scale bar, 100 μm. **B** Effects of miR-485-3p overexpression on the proportion of CD24^+^ CD44^+^ cells in PDAC cells MIA PaCa-2 and PANC-1 under normoxic or hypoxic conditions. **C** Effects of miR-485-3p overexpression on the proportion of CD133^+^ cells in PDAC cells MIA PaCa-2 and PANC-1 under normoxic or hypoxic conditions. **D**, **E** Subcutaneous tumor formation rate of overexpressed miR-485-3p and its control group in nude mice. Data are expressed as mean ± SD from at least three independent experiments. *P < 0.05; **P < 0.01; ***P < 0.001.

Accumulating evidence has revealed that SOX9 is a master regulator of pancreatic progenitor cells and plays an important role in pancreatic endocrine and ductal cell differentiation during pancreatic development [18, 19]. SOX9 is also recognized as a marker of PDAC cancer stem cells (CSCs), contributing to their stemness properties [20]. Therefore, we investigated the role of miR-485-3p in PDAC CSC stemness. Hypoxia enhanced the tumor-sphere formation ability in MIA PaCa-2 and PANC-1, which could be reversed by miR-485-3p overexpression (Fig. 3A). PDAC cells exposed to hypoxia had higher expression of CD24 and CD44 than those exposed to normoxia, while miR-485-3p overexpression inhibited the proportion of CD24^+^CD44^+^ PDAC stem cells (Fig. 3B) and CD133^+^ cells (Fig. 3C). In addition, overexpression of miR-485-3p downregulated the expression of stemness marker SOX2 in MIA PaCa-2 (Supplementary Fig. 5A) and PANC-1 (Supplementary Fig. 5B).

In vivo, miR-485-3p overexpression in PANC-1 decreased the tumor-initiating cell frequency to nearly 1/16 (from 1/25140 to 1/ 417215), which indicated that the frequency of CSCs in the miR-485-3p overexpression group was significantly lower than that in the control group (Fig. 3D). These data suggested that hypoxia increases PDAC cell stemness by suppressing miR-485-3p.

### miR-485-3p promoted ferroptosis

**Fig. 4:**
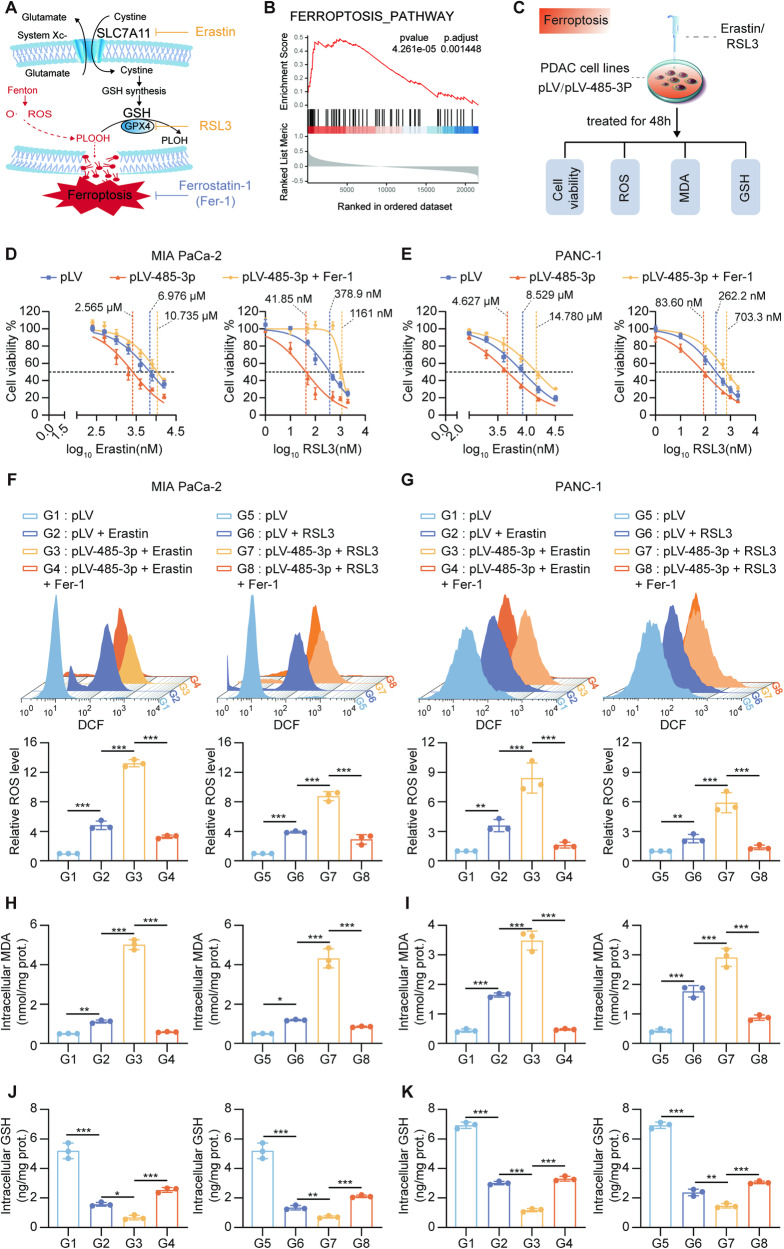
miR-485-3p promotes ferroptosis. **A** Schematic illustration of SLC7A11-GPX4 mediated ferroptosis pathway. **B** GSEA showed that differentially expressed genes identified following SLC7A11 high and low (median) in TCGA-PDAC were enriched in the ferroptosis pathway. **C** The schematic illustration for human PDAC cells ferroptosis evaluation. **D**, **E** Sensitivity to ferroptosis inducers Erastin and RSL3 of MIA PaCa-2 (**D**) and PANC-1 (**E**) overexpressing miR-485-3p. **F**–**K** changes of intracellular ROS (**F**, **G**), MDA (**H**, **I**), and GSH (**J**, **K**) levels in MIA PaCa-2 (**F**, **H**, **J**) and PANC-1 (**G**, **I**, **K**), treated with miR-485-3p combined with ferroptosis inducers (Erastin or RSL3) and or Fer-1. Data are expressed as mean ± SD from at least three independent experiments. *P < 0.05; **P < 0.01; ***P < 0.001.

SLC7A11 is involved in ferroptosis, a form of iron-dependent programmed cell death characterized by intracellular lipid peroxidation induced by excess oxygen free radicals generated via the Fenton reaction during iron metabolism. One essential component of the classic antioxidant systems is the glutathione antioxidant system mediated by the SLC7A11-GPX4 axis (Fig. 4A). In PDAC tissues from TCGA, SLC7A11 expression was correlated with the ferroptosis pathway (Fig. 4B). Therefore, we hypothesized that miR-485-3p may regulate ferroptosis pathway. Changes in the sensitivity to ferroptosis inducers, ROS, MDA, and intracellular GSH levels, as well as the restoration of these indicators by ferroptosis inhibitors, serve as markers for ferroptosis regulation (Fig. 4C).

MiR-485-3p overexpression increased the sensitivity of MIA PaCa-2 and PANC-1 cells to ferroptosis inducers Erastin and RSL3, and this effect was attenuated by ferroptosis inhibitors (Fig. 4D, E). Additionally, ferroptosis inducers significantly elevated intracellular ROS (Fig. 4F, G) and MDA (Fig. 4H, I) levels in PDAC cells overexpressing miR-485-3p, while ferroptosis inhibitor Fer-1 effectively reduced ROS and MDA levels. Furthermore, intracellular glutathione content significantly decreased upon miR-485-4p overexpression, but could be restored by Fer-1 (Fig. 4J, K). These findings suggested that miR-485-3p plays a role in regulating ferroptosis.

### miR-485-3p regulates stemness and chemosensitivity of PDAC cells through SLC7A11-mediated ferroptosis

**Fig. 5:**
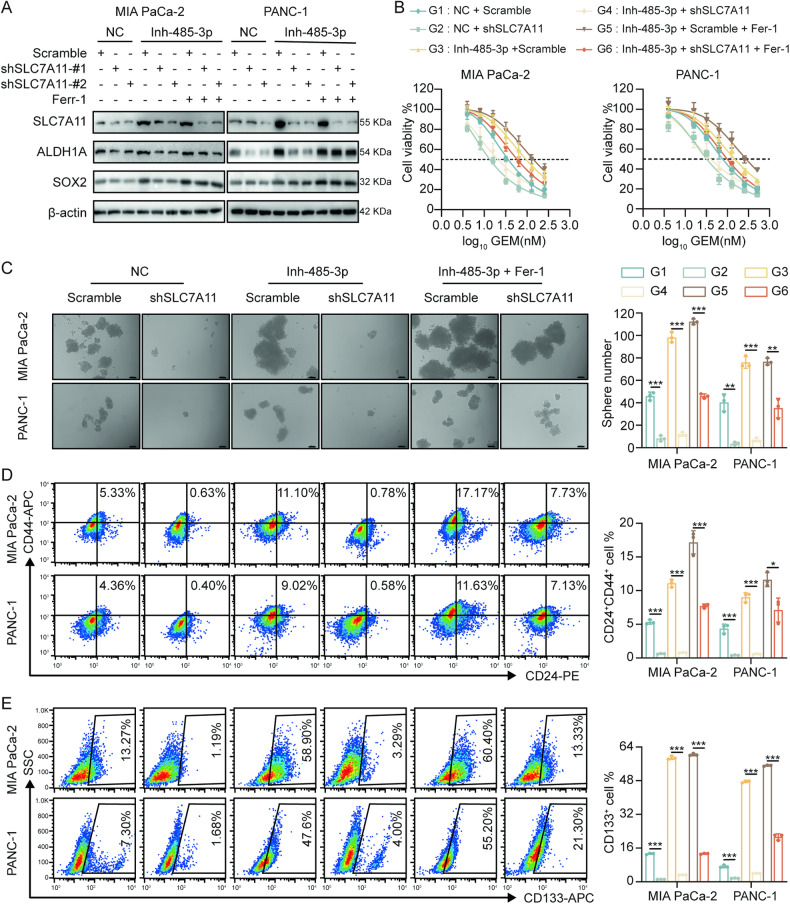
miR-485-3p regulated stemness and chemosensitivity of PDAC cells through SLC7A11-mediated ferroptosis. **A** Expression levels of PDAC CSCs markers in cells with knockdown of miR-485-3p and/or SLC7A11, combined with the treatment with ferroptosis inhibitor Fer-1. **B** The chemosensitivity of PDAC cells with knockdown of miR-485-3p and/or SLC7A11, combined with treatment with Fer-1. **C** The stemness and sphere formation of PDAC cells MIA PaCa-2 and PANC-1 with knockdown of miR-485-3p and/or SLC7A11, combined with treatment with Fer-1. Scale bar, 100 μm. **D** The proportion of CD24 + CD44 stem cells in PDAC cells MIA PaCa-2 and PANC-1 with knockdown of miR-485-3p and/or SLC7A11, combined with treatment with Fer-1. **E** The proportion of CD133^+^ stem cells in PDAC cells MIA PaCa-2 and PANC-1 with knockdown of miR-485-3p and/or SLC7A11, combined with treatment with Fer-1. Data are expressed as mean ± SD from at least three independent experiments. *P < 0.05; **P < 0.01; ***P < 0.001.

The expression of stemness markers SOX2 and ALDH1A significantly increased in response to miR-485-3p silencing in MIA PaCa-2 and PANC-1 cells, while SLC7A11 knockdown significantly attenuated their expression. Treatment with ferroptosis inhibitors significantly upregulated the expression of stemness markers (Fig. 5A). Knockdown of SLC7A11 inhibited drug resistance induced by miR-485-3p silencing, and these effects were restored by Fer-1 (Fig. 5B, Supplementary Fig. 6A, B). Notably, silencing miR-485-3p in MIA PaCa-2 and PANC-1 cells led to an increase in tumor cell sphere formation, while knockdown of SLC7A11 reversed this effect (Fig. 5C). Furthermore, treatment with a ferroptosis inhibitor partially restored the stemness sphere formation inhibited by miR-485-3p knockdown (Fig. 5C). Flow cytometry showed that silencing miR-485-3p increased the proportion of CD24^+^CD44^+^ cells, which was mitigated by SLC7A11 knockdown (Fig. 5D). A similar trend was observed for CD133^+^ cells (Fig. 5E).

Furthermore, miR-485-3p inhibition decreased MDA levels (Supplementary Fig 7A, B) and increased GSH concentration (Supplementary Fig 7C, D), and these effects were reversed by SLC7A11 knockdown and subsequently restored by Fer-1. These results suggested that miR-485-3p regulates PDAC stemness partially through the SLC7A11-mediated ferroptosis pathway.

### The methylation of the miR-485-3p promoter region was mediated by DNMT3B under hypoxia

**Fig. 6:**
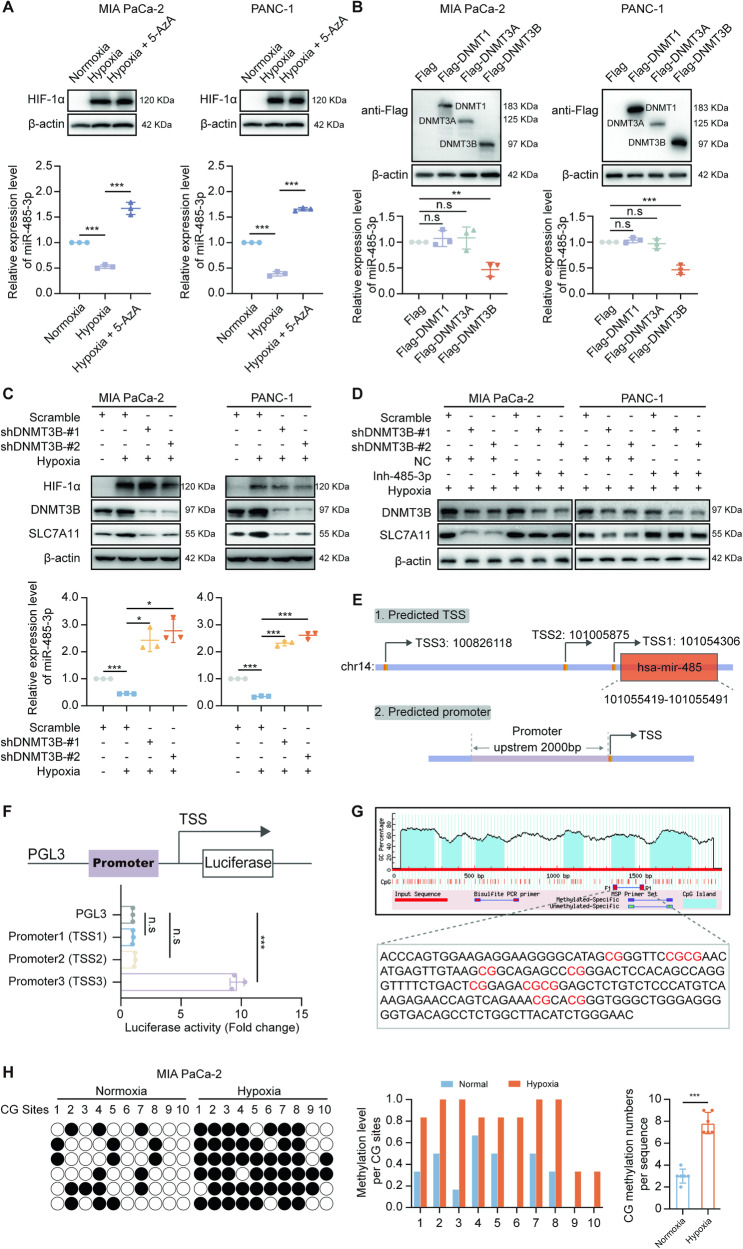
The methylation of the miR-485-3p promoter region was mediated by DNMT3B under hypoxia. **A** The expression of miR-485-3p in cells exposed to hypoxia and/or treated with 5-AZA. **B** The expression of miR-485-3p in cells with the overexpression of DNMTs. **C** The expression of miR-485-3p in cells with knockdown of DNMT3B. **D** The expression of SLC7A11 in cells exposed to hypoxia and/or with the knockdown of DNMT3B and/or treatment with miR-475-3p inhibitor. **E** Online prediction of the possible promoter region of miR-485-3p. **F** Dual-luciferase reporter assay of transcription activity of the three promoter regions. **G** Methprimer predicts the distribution of CpG islands in the promoter region. **H** BSP identification of the CpG methylation level in the miR-485-3p promoter of the intracellular genome after hypoxia exposure, the black circle represents the presence of methylation modification, and the white circle represents the absence of methylation modification in the CpG where it is located. Data are expressed as mean ± SD from at least three independent experiments. *P < 0.05; **P < 0.01; ***P < 0.001.

To explore the molecular mechanism by which hypoxia downregulates miR-485-3p expression in PDAC cells, we considered the role of DNA methylation in regulating the transcription of non-coding RNA. In PDAC cells treated with the DNA methyltransferase inhibitor 5-azacytidine (5-AZA), miR-485-3p expression was restored in hypoxic condition (Fig. 6A). Subsequently, we overexpressed DNMTs in MIA PaCa-2 and PANC-1 cells and found that DNMT3B significantly inhibited miR-485-3p expression (Fig. 6B). PDAC cells with DNMT3B knockdown exhibited lower expression of SLC7A11 compared to scramble group under hypoxia (Fig. 6C). Hypoxia-induced upregulation of SLC7A11 expression was antagonized by DNMT3B knockdown, suggesting that hypoxia elevates SLC7A11 expression by promoting DNMT3B expression (Fig. 6C). Furthermore, miR-485-3p inhibitor could upregulate SLC7A11 expression, indicating that DNMT3B regulates SLC7A11 expression through miR-485-3p (Fig. 6D).

We used TransmiR (www.cuilab.cn/transmir), mirTrans (mcube.nju.edu.cn/jwang/lab/soft/mirtrans/) and geneXplain (platform.genexplain.com/) to screen regions of miR-485-3p promoter and identified three possible sites responsible for miR-485-3p transcription (Fig. 6E). Promoter3 showed strong transcriptional activity, suggesting that Promoter3 may contain the promoter region for miR-485-3p (Fig. 6F). Methprimer tool predicted CpG island in this region (Fig. 6G). We found a higher level of methylation in the miR-485-3p promoter region in cells exposed to hypoxia compared to cells exposed to normoxia (Fig. 6H). Furthermore, we generated the DNMT3B flag-tagged vector and induced exogenous expression in MIA PaCa-2 and PANC-1 PDAC cell lines, followed by chromatin immunoprecipitation assay. Our results confirmed that DNMT3B could directly bind to the promoter of miR-485-3p and transcriptionally regulated miR-485-3p level (Supplementary Fig. 8A, B). Thus, we provide evidence here that DNMT3B is a major negative regulator of miR-485-3p expression in PDAC cells, especially under hypoxic conditions (Supplementary Fig. 8A, B). These data indicated that the miR-485-3p promoter is methylated by DNMT3B under hypoxic conditions, resulting in miR-485-3p downregulation and subsequent upregulation of SLC7A11 expression.

### DNMT3B regulates stemness and chemosensitivity through SLC7A11-mediated ferroptosis in PDAC cells

**Fig. 7:**
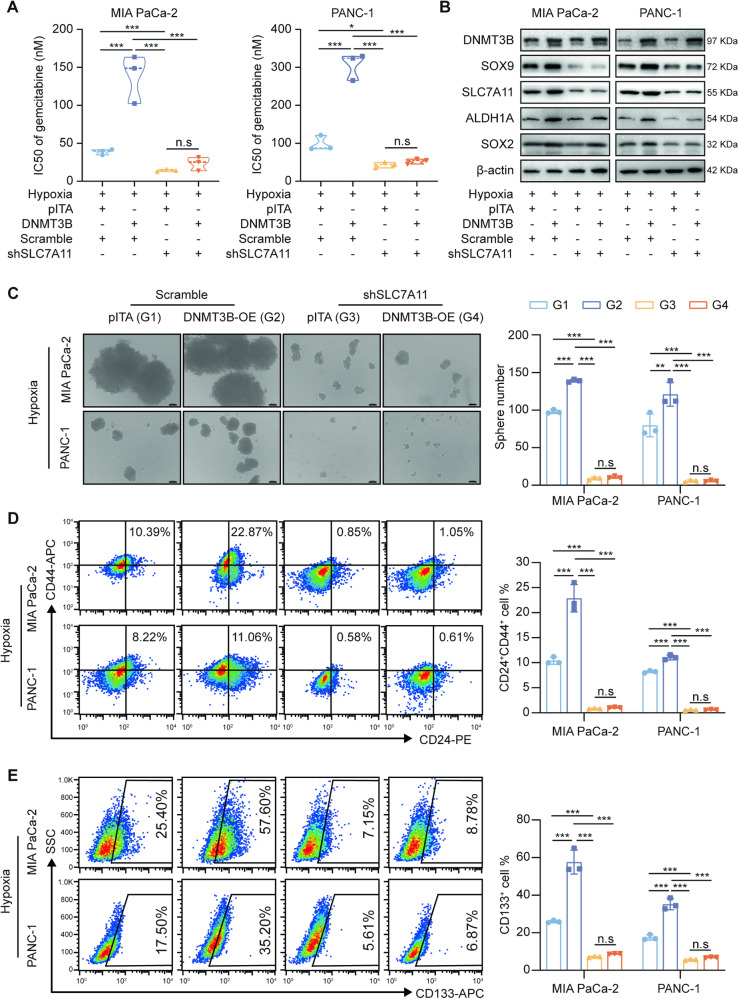
DNMT3B-SLC7A11 regulates stemness and chemosensitivity in PDAC cells. **A** IC50 of gemcitabine in MIA PaCa-2 and PANC-1 with DNMT3B overexpression and/or SLC7A11 knockdown under hypoxia condition. **B** The expression levels of stem cell markers in PDAC cells with DNMT3B overexpression and/or SLC7A11 knockdown under hypoxia conditions. **C** Sphere formation of PDAC cells with DNMT3B overexpression and/or SLC7A11 knockdown under hypoxia condition. Scale bar, 100 μm. **D** The proportion of CD24^+^CD44^+^ cells in PDAC cells with DNMT3B overexpression and/or SLC7A11 knockdown under hypoxia condition. **E** The proportion of CD133^+^ cells in PDAC cells with DNMT3B overexpression and/or SLC7A11 knockdown under hypoxia condition. Data are expressed as mean ± SD from at least three independent experiments. *P < 0.05; **P < 0.01; ***P < 0.001.

To confirm whether DNMT3B plays an oncogenic role in PDAC cells by upregulating SLC7A11 expression and inhibiting the ferroptosis process, we assessed the effect of DNMT3B overexpression and SLC7A11 knockdown on PDAC cell sensitivity to gemcitabine. Overexpression of DNMT3B reduced the sensitivity of PDAC cells to gemcitabine, while SLC7A11 knockdown significantly reversed this effect (Fig. 7A). Similar changes were observed for stemness marker expression (Fig. 7B). PDAC cells with both DNMT3B overexpression and SLC7A11 silencing had significantly less tumor-sphere formation compared to cells with only DNMT3B overexpression (Fig. 7C). Meanwhile, overexpression of DNMT3B under hypoxic condition increased the proportion of CD24^+^CD44^+^ (Fig. 7D) and CD133^+^ cells (Fig. 7E). However, SLC7A11 knockdown reduced the proportion of CD24^+^CD44^+^ and CD133^+^ cells. These data indicate that SLC7A11 knockdown inhibits stemness maintenance induced by DNMT3B.

We further examined biochemical indicators of ferroptosis, such as ROS, MDA, and intracellular GSH content (Supplementary Fig. 9A). PDAC cells exposed to gemcitabine, DNMT3B overexpression significantly inhibited ACSL4 expression, reduced ROS and MDA levels and increase GSH levels (Supplementary Fig. 9B–F). Silencing SLC7A11 could reverse these effects, which was then partially restored by Fer-1 (Supplementary Fig. 9B–F). These results demonstrate that ferroptosis mediated by DNMT3B-SLC7A11 axis regulates the levels of ROS and lipid peroxidation in PDAC cells exposed to gemcitabine.

### miR-485-3p sensitizes PDAC cells to gemcitabine mediated by ferroptosis in vivo and DNMT3B is correlated with SLC7A11 expression in PDAC patients

**Fig. 8:**
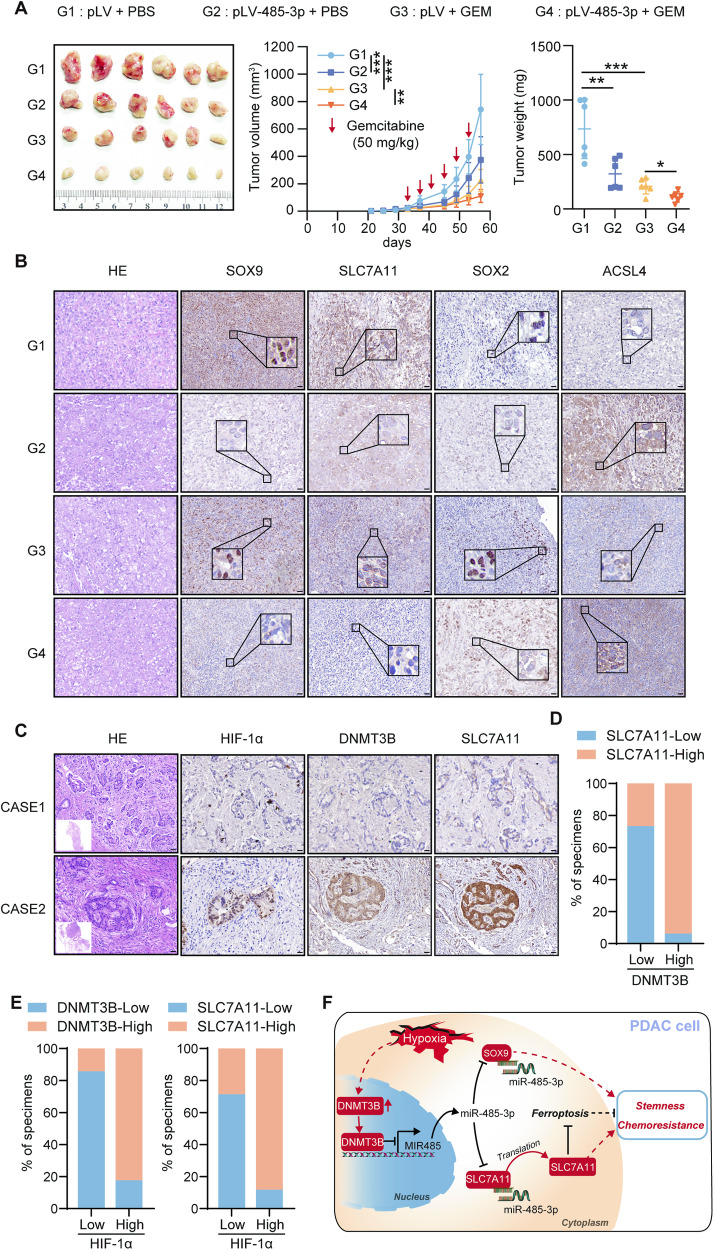
MiR-485-3p increased chemosensitivity in vivo and the correlation between DNMT3B and SLC7A11 expression in patients with pancreatic cancer. **A** Tumor formation in groups of PDAC cell lines with control or miR-485-3p overexpression after treatment with gemcitabine. The volume growth and tumor weight of subcutaneous tumors were recorded. **B** The levels of SOX9, SLC7A11, SOX2, and ACSL4 were assessed in different groups by IHC. Scale bar, 50 μm. **C** Representative IHC staining for HIF-1α, DNMT3B, and SLC7A11 in PDAC patients. CASE1 and CASE2 refer to two representative samples categorized by low and high DNMT3B expression. Scale bar, 50 μm. **D** The percentage of specimens with low or high SLC7A11 expression in the low or high DNMT3B expression groups. **E** The percentage of specimens with low or high DNMT3B and SLC7A11 expression in the low or high HIF-1α expression groups. **F** Graphical abstract highlighting the role of hypoxia/DNMT3B/miR-485-3p/SLC7A11 and SOX9 axis in regulating stemness and chemoresistance of PDAC cells.

To confirm that miR-485-3p sensitizes PDAC cells to gemcitabine in vivo, we found that tumors with miR-485-3p overexpression group grew much slower than those with pLV, and miR-485-3p overexpression enhanced the responsiveness of PDAC to gemcitabine (Fig. 8A). Moreover, miR-485-3p overexpression inhibited SOX9, SLC7A11 and SOX2 expression, while upregulated ACSL4 expression (Fig. 8B). The results indicate that miR-485-3p decreases PDAC cell stemness and increases sensitivity to gemcitabine mediated by ferroptosis in vivo.

We further assessed HIF-1α, DNMT3B, and SLC7A11 expression by IHC in 31 human PDAC tissues (Fig. 8C). In consistent with the investigations that DNMT3B upregulates SLC7A11 expression in cultured cells, the expression of DNMT3B was positively correlated with SLC7A11 expression. Our results demonstrated that the expression level of SLC7A11 was significantly higher in DNMT3B high-expression group compared to DNMT3B low-expression group (Fig. 8D). Moreover, the expression of HIF-1α positively correlated with DNMT3B and SLC7A11 expression (Fig. 8E). Taken together, the data suggest the important pathological role of DNMT3B-SLC7A11 axis in pancreatic cancer.

## Discussion

In this study, we reported that miR-485-3p was downregulated in PDAC cells exposed to hypoxia and further explored the role of miR-485-3p in PDAC chemoresistance. Overexpression of miR-485-3p markedly decreased the tumor-sphere formation ability and gemcitabine resistance in PDAC cells. In addition, miR-485-3p inhibited PDAC in vivo model. Mechanistically, miR-485-3p downregulated SOX9 and SLC7A11 expression by directly binding to mRNAs and promoting degradation. Furthermore, miR-485-3p regulated the ferroptosis pathway via SLC7A11. We also demonstrated that the miR-485-3p promoter is hypermethylated by DNMT3B, contributing to miR-485-3p aberrant expression under hypoxia. Moreover, DNMT3B-SLC7A11 decreased the tumor-sphere formation ability and gemcitabine resistance in PDAC cells with the regulation of ferroptosis (Fig. 8F).

Hypoxia is a common characteristic of PDAC microenvironment, with a median tissue partial oxygen pressure (pO2) of 0–5.3 mmHg (0–0.7%) compared to the adjacent normal pancreas pO2 at 24.3–92.7 mmHg (3.2–12.3%) [21]. Hypoxic areas within tumors provide well-established niches for cancer cells to acquire and maintain stem-like properties, referred to as CSC phenotype [4]. Pancreatic CSCs harbor high heterogeneity in terms of surface and intracellular markers, such as CD24, CD44, CD133, SOX2, and ALDH1A, as well as their response to chemotherapy and hypoxia [4, 22]. Besides, Chen et al. demonstrated that HIF-1α induced by P4HA1 contributed to PDAC stemness and chemoresistance [23]. However, few studies have determined the mechanisms by which miRNAs regulate the stemness and chemoresistance of PDAC. Our discovery that miR-485-3p targets SOX9 and SLC7A11 to inhibit stemness-like properties and gemcitabine resistance provides new insights into the regulation of hypoxia, CSCs, and chemoresistance in pancreatic cancer.

SLC7A11 is a key component of the cystine/glutamate transporter and plays an important role in cell metabolism and ferroptosis regulation [24]. Ferroptosis is a form of regulated cell death characterized by iron-dependent lipid peroxidation. SLC7A11 negatively regulates ferroptosis by facilitating cystine uptake, which, in turn, leads to glutathione synthesis and scavenging of reactive oxygen species [24]. Ferroptosis has been implicated in stemness maintenance and chemotherapy resistance in various cancers and is an attractive target for therapeutic intervention [25, 26]. Targeting SLC7A11 can inhibit the proliferation ability of various tumor cells such as colorectal cancer, breast cancer, and pancreatic cancer. MiR-375 inhibited the stemness characteristics of gastric cancer cells by targeting SLC7A11-mediated ferroptosis [9]. SLC7A11-mediated ferroptosis in glioma cells was involved in regulating the stemness maintenance of glioma cells [27]. In addition, ferroptosis significantly inhibited the growth of PDAC in SLC7A11-deficient genetically engineered mice [28]. However, the role of SLC7A11 in PDAC CSCs remains unclear. In this study, we found that miR-485-3p directly targets and significantly inhibits the expression of SLC7A11. Furthermore, silencing miR-485-3p promoted stemness marker expression and sphere formation. Importantly, these effects could be attenuated by ferroptosis inhibitor Fer-1. These results suggest that miR-485-3p may modulate stemness maintenance and chemotherapy resistance through the SLC7A11-mediated ferroptosis pathway.

Notably, significant downregulation of miR-485-3p in PDAC tissues was correlated with poor patient prognosis, consistent with previous studies identifying miR-485-3p as a tumor suppressor in breast cancer, colorectal cancer, and prostate cancers [15, 16, 29]. However, the mechanism of miR-485-3p downregulation in PDAC cells, especially under hypoxia, is still unclear. DNA methylation, one of epigenetic modifications, plays a central role in regulating gene expression [30, 31]. We found that hypoxia in PDAC cells led to the upregulation of DNMT3B, which subsequently promoted the hypermethylation of the miR-485-3p promoter region. The epigenetic modification resulted in reduced miR-485-3p expression and, in turn, upregulated the expression of SOX9 and SLC7A11, contributing to stemness maintenance and gemcitabine resistance. The results of the chromatin immunoprecipitation assay confirmed that DNMT3B could directly bind to the promoter of miR-485-3p in PDAC cells under hypoxia, which indicates that the miR-485-3p promoter might be inaccessible to DNMT3b in normoxia due to specific chromatin modifications or binding of other regulatory factors.

This study has several limitations. First, while our findings strongly suggest that the identified region is the promoter region of miR-485-3p, we did not confirm it. Chromatin immunoprecipitation or reporter gene assays are needed to provide conclusive evidence. Second, while we found a positive correlation between HIF-1α and DNMT3B expression, the detailed mechanisms require deeper exploration. Dissection of the pathways that modulate DNMT3B expression in hypoxic environments could shed light on potential therapeutic targets.

## Conclusion

In summary, miR-485-3p is downregulated in PDAC tissues and cells, and its aberrant expression predicts a poor prognosis in PDAC patients. MiR-485-3p acts as a tumor suppressor to inhibit stemness and gemcitabine resistance in PDAC cells. Mechanistically, the region of the miR-485-3p promoter is hypermethylated by DNMT3B induced by hypoxia, resulting in the upregulation of SOX9 and SLC7A11 to hinder the ferroptosis process.

## Materials and methods

### Tissue specimens

A total of 31 PDAC tissue samples, along with their matched adjacent non-PDAC tissues, were retrospectively collected from patients who underwent R0 resection at Peking University First Hospital from January 2013 to December 2017 and with comprehensive follow-up data. This study was conducted in accordance with the Declaration of Helsinki and approved by the local ethics committee of Peking University First Hospital (No. 201933) [17].

### Cell culture, plasmid construction, and transfection

Human PDAC cell lines, including Mia PaCa-2, PANC-1, and 293 T, were cultured in the general surgery laboratory of Peking University First Hospital and tested for mycoplasma contamination. Gemcitabine-resistant (GR) cell lines Mia PaCa-2 and PANC-1 were maintained in a complete medium containing 200 nM gemcitabine (Gemzar, Eli Lilly). Lentivirus plasmids pLV-485-3p and pLV-Inh-485-3p were purchased from GenePharma (Shanghai, China). miR-485-3p mimics (miR10002176) and inhibitors (miR20002176) were obtained from Ribobio (Guangzhou, China). Lentiviral vectors for SLC7A11 shRNA or DNMT3B shRNA were constructed based on the Plko.1 plasmid. Human DNMT1, DNMT3A, and DNMT3B coding sequences with flag-tags, amplified from a cDNA library, were cloned into a pITA vector. Sequences for cloning are detailed in Supplementary Table S1. Transfection was performed following the Vigofect (Vigorous Biotechnology, Beijing, China) instructions.

### q-PCR and western blot analysis

Total RNA from cells was extracted using the TRIzol kit (Invitrogen, #15596018, USA). Reverse transcription into cDNA was accomplished using a SYBR Kit (Vazyme Biotech, #P611, Nanjing, China). The 7500 equipment was employed to measure mRNA expression levels of target genes normalized to β-actin (ACTB). qPCR primer sequences are listed in Supplementary Table S2.

Cells were lysed on ice for 30 min using cell lysis buffer (Beyotime, China) supplemented with proteinase inhibitor Cocktail (Roche, USA). The lysateds were centrifuged at 4 °C (12,000 × *g*, 10 min) and proteins were separated by SDS-PAGE (Biotides, China) and transferred to PVDF membranes (Bio-Rad, USA). The membranes were blocked with 5% skim milk powder in TBST and primary antibodies anti-HIF-1α (Abcam, #ab279654, USA), anti-SOX9 (Abclonal, #A19710, China), anti-SLC7A11 (Abclonal, #A2413, China), anti-ALDH1A (Abclonal, #A0517, China), anti-SOX2 (Abcam, #Ab92494, USA), anti-β-actin (MBL, #PM053, Japan), anti-ACSL4(Abclonal, #A20414, China), anti-flag HRP (Abmart, #PA9020), and anti-DNMT3B (Abclonal, #A7239, China) at 4 °C overnight. The membranes were washed with TBST for 5 min (3 times) and incubated with HRP-labeled goat anti-rabbit IgG(H + L) (Earthox, #E030120, USA) or -mouse IgG(H + L) (Earthox, #E030110) for 40 min. ECL substrate (Millipore, #WBULS0500, USA) was applied to develop the membranes.

### In vivo tumorigenicity assay

BALB/c nude mice were randomized into different groups. Cancer cells at different dilutions were subcutaneously injected into the flanks of the mice (*n* = 10) using a random number method with no blinding. Stem cell frequency was calculated using the website (http://bioinf.wehi.edu.au/software/elda/).

For in vivo tumor estimation, a total of 1 × 10^7^ PANC-1 cells were inoculated subcutaneously into the right flanks of nude mice (*n* = 6) using the random number method with no blinding. The mice were treated with 50 mg/kg gemcitabine or equal volumes of PBS. The tumor size was calculated and the mice were sacrificed at the indicated time. The resected tumor was kept in liquid nitrogen.

### Tumor-sphere formation assay

Cells were cultured in ultralow adhesion plates at 1000 cells/ per well in serum-free medium with B27 (1:50), EGF (20 ng/ml), bFGF (100 ng/ml), and LIF (10 ng/ml). After culture for 2 weeks, tumor spheres with a diameter of >100 μm were counted.

### CCK-8 assay

Cells were seeded into 96-well plates (3000 cells/well) with 100 μl medium containing 10% FBS and treated with gemcitabine for 72 h or RSL3 or Erastin for 48 h. Next, cells were treated with 100 μl of DMEM and CCK-8 (Dojindo, Japan) mixture (90 μl:10 μl) at 37 °C for 1 h, and absorbance was measured at 450 nm using a 96-well plate reader.

### Flow cytometry

Cells were stained with anti-hCD24 (Biolegend, #311118, USA), anti-hCD44(Biolegend, #338816, USA), anti-hCD133(Biolegend, #393906, USA), or appropriate control antibodies, with isotype controls used as negative controls or ROS probes DCFH-DA (Beyotime, #S0033S, China). Data analysis was performed using FlowJo V.10.0.

### Luciferase assay

Following transfection of luciferase reporter plasmids and mutant sequences containing the 3’-UTR of SLC7A11 and SOX9 with miR-485-3p binding sites into cells for 24 h, Dual-Luciferase® Reporter Assay System (Promega, E1910) was employed to measure firefly luciferase activity and renilla luciferase activity. The ratio of the two was calculated and compared with the control group to determine relative luciferase activity.

### Malondialdehyde (MDA) assay

The level of MDA was evaluated using the MDA detection kit (Beyotime, #S0131S, China) following the instructions.

### GSH assay

The reduced glutathione content was evaluated by using the reduced glutathione detection kit (Solarbio, BC1170, China) following the instructions.

### Bisulfite sequencing PCR (BSP)

Genomic DNA was extracted from cells using the DNA extraction Kit (TIANGEN, #DP304-02, China). DNA was denatured by incubation with NaOH for 10 min at 37 °C, followed by bisulfite modification, PCR amplification, cloning to the pGM-T vector (TIANGEN, #VT402 and #VT202-01, China), and then sequencing by Beijing Tsingke Biotech Company.

### CHIP assay

ChIP was performed with lysates prepared from MIA PaCa-2 and PANC-1 with Flag-DNMT3B and Flag overexpression, then measured by using EpiQuik^TM^ Chromatin Immunoprecipitation Kit (Epigentek, P-2002). Anti-Flag-Tag mAb (AE121) was purchased from Abclonal, SYBR Green qRT-PCR was performed using the human DNMT3B primers (Supplementary Table S2).

### Immunohistochemistry

IHC scoring was conducted by two independent pathologists who were blinded to patients’ clinicopathological features and prognosis.

### Bioinformatic analysis

The differential expression genes (DEGs) of gemcitabine-sensitive and -resistant pancreatic cancer cells in GEO datasets were analyzed by GEO2R tool (https://www.ncbi.nlm.nih.gov/geo/geo2r). KEGG enrichment of DEGs was analyzed by DAVID online tool (https://david.ncifcrf.gov/tools.jsp) and visualized by the bioinformatics tool (https://www.bioinformatics.com.cn). The Starbase database (https://starbase.sysu.edu.cn/) was used to predict the targets of miR-485-3p.

### Statistical analysis

Data were analyzed using SPSS 27.0 software. For continuous variables with homogeneous variances, a *t*-test was performed, and results were presented as the mean ± SD. *χ*^2^ test was used for categorical variables. Unpaired Student’s *t*-test was used for in vivo experiments. Survival analysis was conducted using Kaplan–Meier survival curves and Log-rank tests. The difference was considered statistically significant at *P* < 0.05.

### Supplementary information

Supplementary Table S1

Supplementary Table S2

Supplementary Figure S1

Supplementary Figure S2

Supplementary Figure S3

Supplementary Figure S4

Supplementary Figure S5

Supplementary Figure S6

Supplementary Figure S7

Supplementary Figure S8

Supplementary Figure S9

Supplementary figure legends

Supplementary table legends

Full and uncropped western blots

## Data Availability

The original contributions presented in the study are included in the article/Supplementary Material; further inquiries can be directed to the corresponding author.
