# Supplementary material for: Hypoxia-induced epigenetic regulation of miR-485-3p promotes stemness and chemoresistance in pancreatic ductal adenocarcinoma via SLC7A11-mediated ferroptosis
Source: Cell Death Discov. 2024 May 29;10:262. doi: 10.1038/s41420-024-02035-x (PMC11137092; doi:10.1038/s41420-024-02035-x)
Supplement: Supplementary file 1 — Supplementary Table S1 [file 41420_2024_2035_MOESM1_ESM.docx]

**Supplementary Table S1.** The sequences of lentivirus or plasmids used in this study.

| **Primers for SLC7A11- and SOX9-3’UTR luciferase reporter** | |
| --- | --- |
| SLC7A11-3’utr-wt1-F | TGTTTAAACGAGCTCTAAATTTCCTACTTAAGTCTTAAGAACTGGG |
| SLC7A11-3’utr-wt1-R | TGCCTGCAGGTCGACTGTCTTGCCTTTTTCTGACATGTG |
| SLC7A11-3’utr-wt2-F | TGTTTAAACGAGCTCATGTGTGTGTTTCAGCGTACTTGAATC |
| SLC7A11-3’utr-wt2-R | CATGCCTGCAGGTCGACTAATAGGTTTTCCCACAAGATGCTGTAATAATACC |
| SLC7A11-3’utr-wt3-F | TGTTTAAACGAGCTCGTTTCCCCAATTACAATTTGACATATC |
| SLC7A11-3’utr-wt3-R | CATGCCTGCAGGTCGACTCAGGACCTCGAATGGAACATG |
| SOX9-3’utr-wt-F | TGTTTAAACGAGCTCGCAGCGAAATCAACGAGAAAC |
| SOX9-3’utr-wt-R | CATGCCTGCAGGTCGACACAATTCTGTTTTTAAATGTTAATAC |
| SLC7A11-3’utr-mut1-F | TTTTCACTAGAACTTCTCAACATTTGGGAACTTTGCAAATATGA |
| SLC7A11-3’utr-mut1-R | TGAGAAGTTCTAGTGAAAACAGTATGTATTGTGCAAAGATGAAAATTTGGAGCCAATGTC |
| SLC7A11-3’utr-mut2-F | CAAGTGAATTAATGTATTGGTTAGGAGAACTGCTTGCTAAG |
| SLC7A11-3’utr-mut2-R | ACATTAATTCACTTGAAACAGTATGTTTTAAAAAAAATCAGTAGTGTATATGTTGAGGAAAAAAAG |
| SLC7A11-3’utr-mut3-F | GATAAAAAACCGAAGGCCAGAGAATCAGGAAATGAAAG |
| SLC7A11-3’utr-mut3-R | CTTCGGTTTTTTATCTTCAGTATGCTTATACCTCAATTACAAAAGATCAACTTTAACAAAGGTC |
| SOX9-3’utr-mut-F | CATTACCATTTTGAGGGGATTTATACATATTTTTAGA |
| SOX9-3’utr-mut-R | CTCAAAATGGTAATGAAAGTATGTGCCGAGAAGGAAAAAATATTTAAAATAGAGAATATTCCTC |
| **Primers for shRNA plasmids** | |
| pLKO.1 SLC7A11-sh1-F | CCGGGCAGCTACTGCTGTGATATCCCTCGAGGGATATCACAGCAGTAGCTGCTTTTTG |
| pLKO.1 SLC7A11-sh1-R | AATTCAAAAAGCAGCTACTGCTGTGATATCCCTCGAGGGATATCACAGCAGTAGCTGC |
| pLKO.1 SLC7A11-sh2-F | CCGGGCACCCTTTGACAATGATAATCTCGAGATTATCATTGTCAAAGGGTGCTTTTTG |
| pLKO.1 SLC7A11-sh2-R | AATTCAAAAAGCACCCTTTGACAATGATAATCTCGAGATTATCATTGTCAAAGGGTGC |
| pLKO.1 DNMT3B-sh1-F | CCGGCCATGCAACGATCTCTCAAATCTCGAGATTTGAGAGATCGTTGCATGGTTTTTG |
| pLKO.1 DNMT3B-sh1-R | AATTCAAAAACCATGCAACGATCTCTCAAATCTCGAGATTTGAGAGATCGTTGCATGG |
| pLKO.1 DNMT3B-sh2-F | CCGGGCCCGTGATAGCATCAAAGAACTCGAGTTCTTTGATGCTATCACGGGCTTTTTG |
| pLKO.1 DNMT3B-sh2-R | AATTCAAAAAGCCCGTGATAGCATCAAAGAACTCGAGTTCTTTGATGCTATCACGGGC |
| **Primers for DNMTs-overexpression plasmid** | |
| pITA DNMT1-F | CAAGCTCGCGGCCGCGCCACCATGCCGGCGCGTACCGCCCC |
| pITA DNMT1-R | GCGAATTCCTACGTACTACTTATCGTCGTCATCCTTGTAATCGTCCTTAGCAGCTTCCT |
| pITA DNMT3A-F | CAAGCTCGCGGCCGCGCCACCATGCCCGCCATGCCCTCCAG |
| pITA DNMT3A-R | GCGAATTCCTACGTATCACTTATCGTCGTCATCCTTGTAATCGGGTATGCTGGTGGGCC |
| pITA DNMT3B-F | CAAGCTCGCGGCCGCGCCACCATGAAGGGAGACACCAGGCA |
| pITA DNMT3B-R | GCGAATTCCTACGTACTACTTATCGTCGTCATCCTTGTAATCTTCACATGCAAAGTAGT |
| **Primers for predicted promoters of miR-485-3p** | |
| PROMO1-F | CTCGCTAGCCTCGAGGTTGGCTGGAAAGCAAGGGCTTG |
| PROMO1-R | CGGATTGCCAAGCTTCCACAGAAAAGCAGAGACAAGACAGAC |
| PROMO2-F | CTCGCTAGCCTCGAGGCATCCCACCTGAGGCCTGG |
| PROMO2-R | CGGATTGCCAAGCTTGCTAGGGGCTGGCTGGGTGGGGT |
| PROMO3-F | CTCGCTAGCCTCGAGGACAGCGAGACAGGATACTGGCAG |
| PROMO3-R | CGGATTGCCAAGCTTCACAACCGCCCAGGTCATTATCAAG |
