# Supplementary material for: Hypoxia-induced epigenetic regulation of miR-485-3p promotes stemness and chemoresistance in pancreatic ductal adenocarcinoma via SLC7A11-mediated ferroptosis
Source: Cell Death Discov. 2024 May 29;10:262. doi: 10.1038/s41420-024-02035-x (PMC11137092; doi:10.1038/s41420-024-02035-x)
Supplement: Supplementary file 2 — Supplementary Table S2 [file 41420_2024_2035_MOESM2_ESM.docx]

**Supplementary Table S2.** List of primers for qPCR used in this study.

| miR-485-3p -F | CAGTCATACACGGCTCTCCTC |
| --- | --- |
| miR-485-3p -R | CCAGTGCAGGGTCCGAGGT |
| U6-F | CACGCAGTGCTCGCTTCG |
| U6-R | CCAGTGCAGGGTCCGAGGT |
| SLC7A11-F | TCTCCAAAGGAGGTTACCTGC |
| SLC7A11-R | AGACTCCCCTCAGTAAAGTGAC |
| SOX9-F | AGCGAACGCACATCAAGAC |
| SOX9-R | CTGTAGGCGATCTGTTGGGG |
| β-actin-F | CATGTACGTTGCTATCCAGGC |
| β-actin-R | CTCCTTAATGTCACGCACGAT |
| CHIP-DNMT3B-F | CATAGCGGGTTCCGCGAACA |
| CHIP-DNMT3B-R | GGTTCTCTTTGACATGGGAG |
