# Supplementary figures and images for: Hypoxia-induced epigenetic regulation of miR-485-3p promotes stemness and chemoresistance in pancreatic ductal adenocarcinoma via SLC7A11-mediated ferroptosis
Source: Cell Death Discov. 2024 May 29;10:262. doi: 10.1038/s41420-024-02035-x (PMC11137092; doi:10.1038/s41420-024-02035-x)

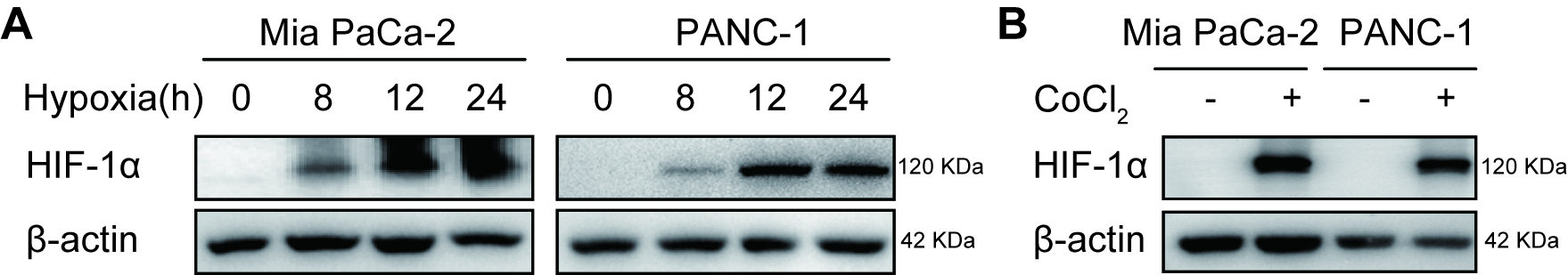

Supplement: Supplementary file 3 — Supplementary Figure S1 [file 41420_2024_2035_MOESM3_ESM.tif]

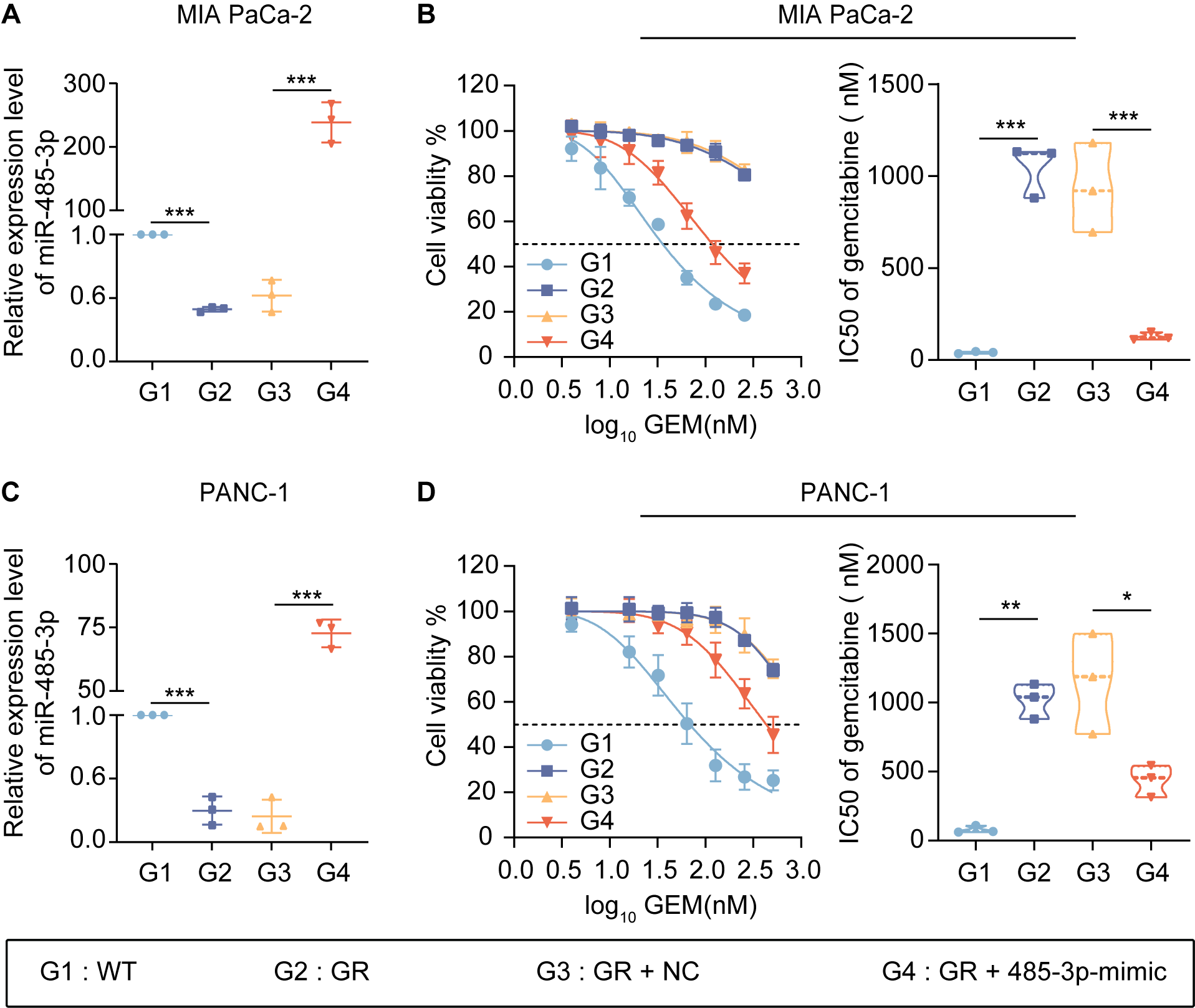

Supplement: Supplementary file 4 — Supplementary Figure S2 [file 41420_2024_2035_MOESM4_ESM.tif]

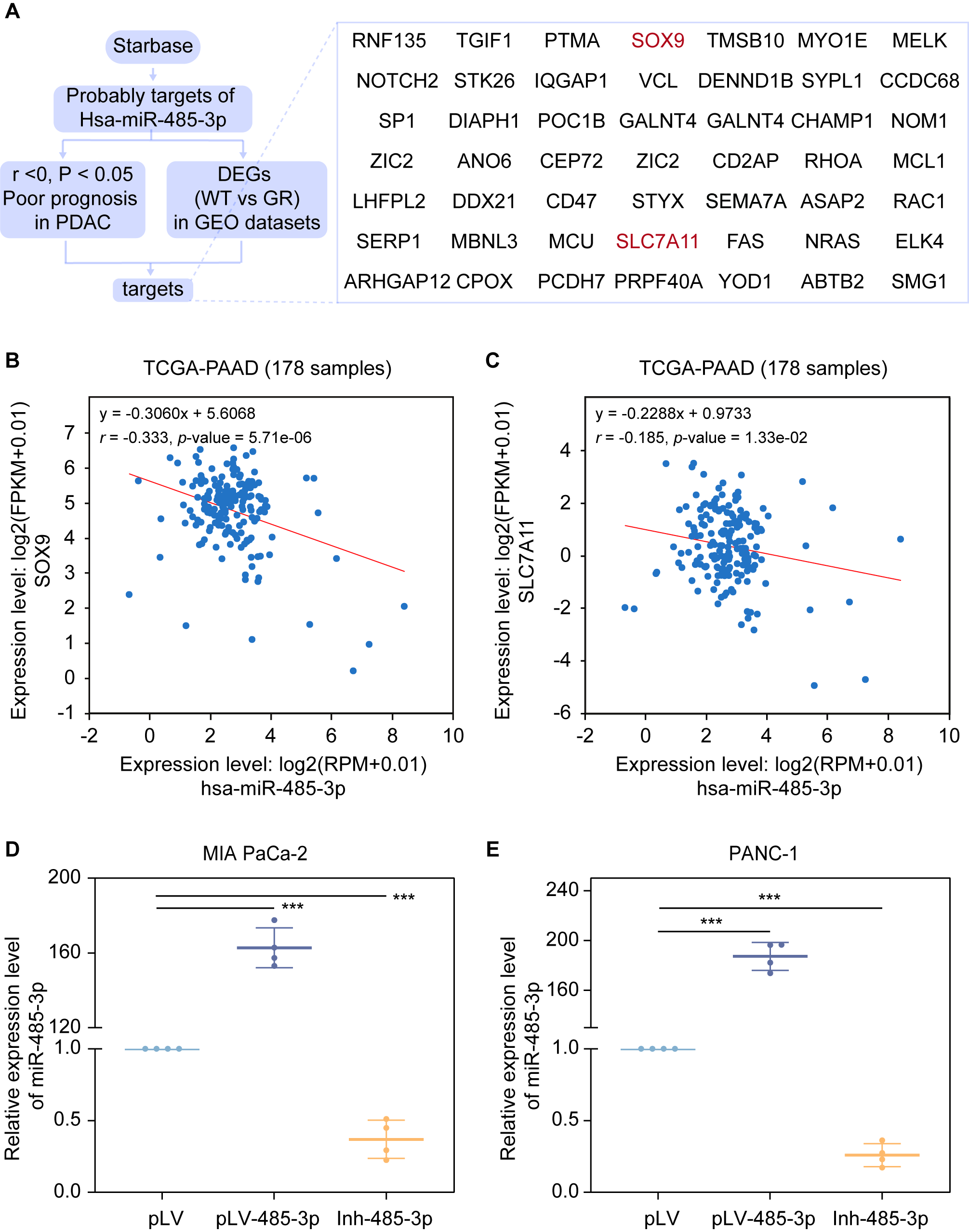

Supplement: Supplementary file 5 — Supplementary Figure S3 [file 41420_2024_2035_MOESM5_ESM.tif]

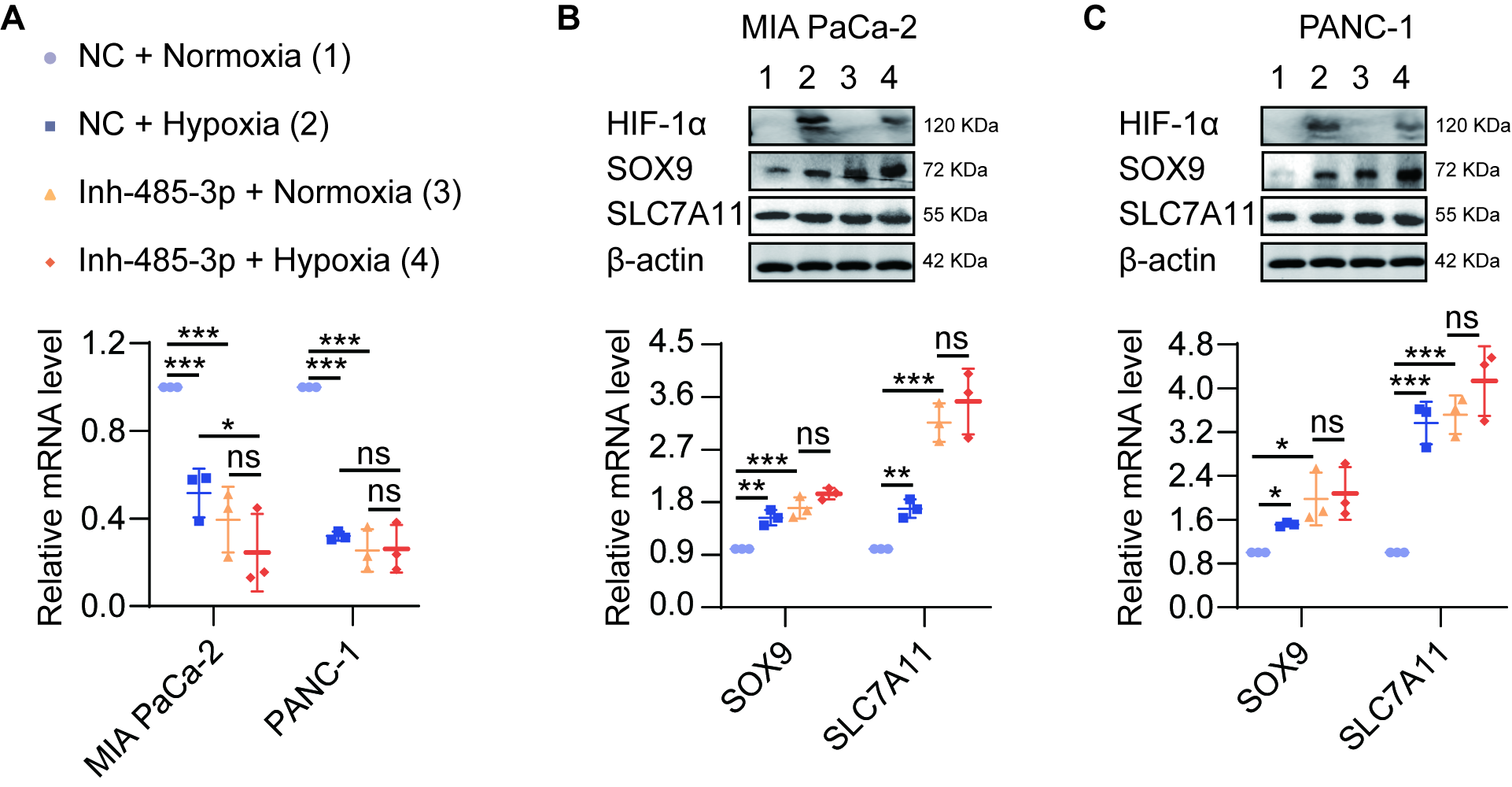

Supplement: Supplementary file 6 — Supplementary Figure S4 [file 41420_2024_2035_MOESM6_ESM.tif]

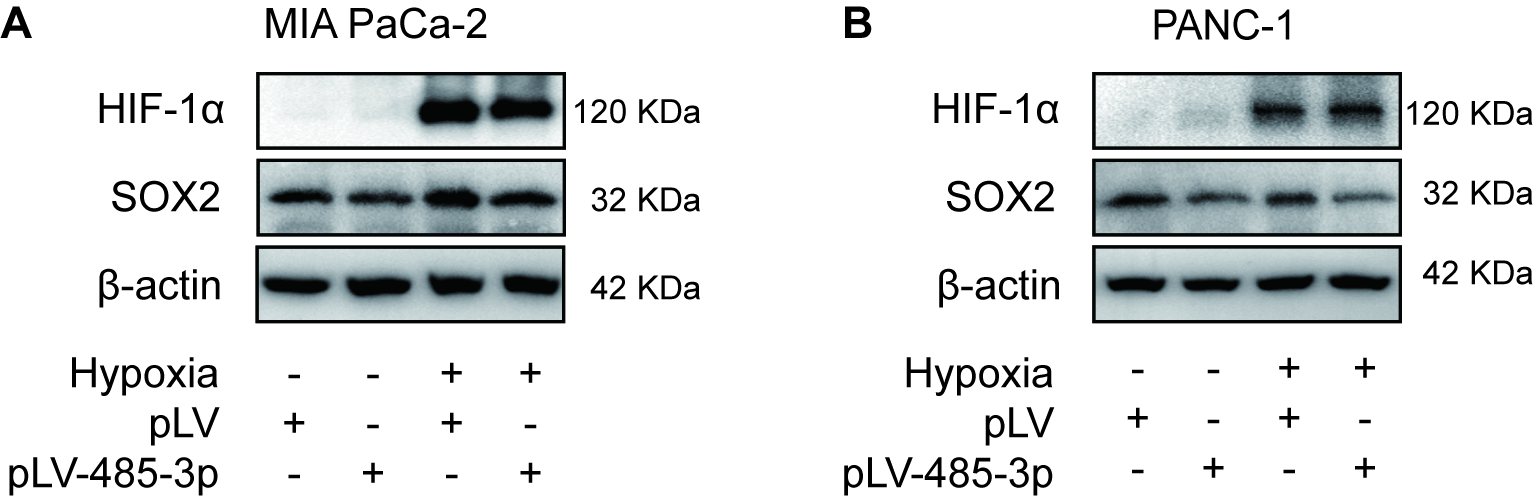

Supplement: Supplementary file 7 — Supplementary Figure S5 [file 41420_2024_2035_MOESM7_ESM.tif]

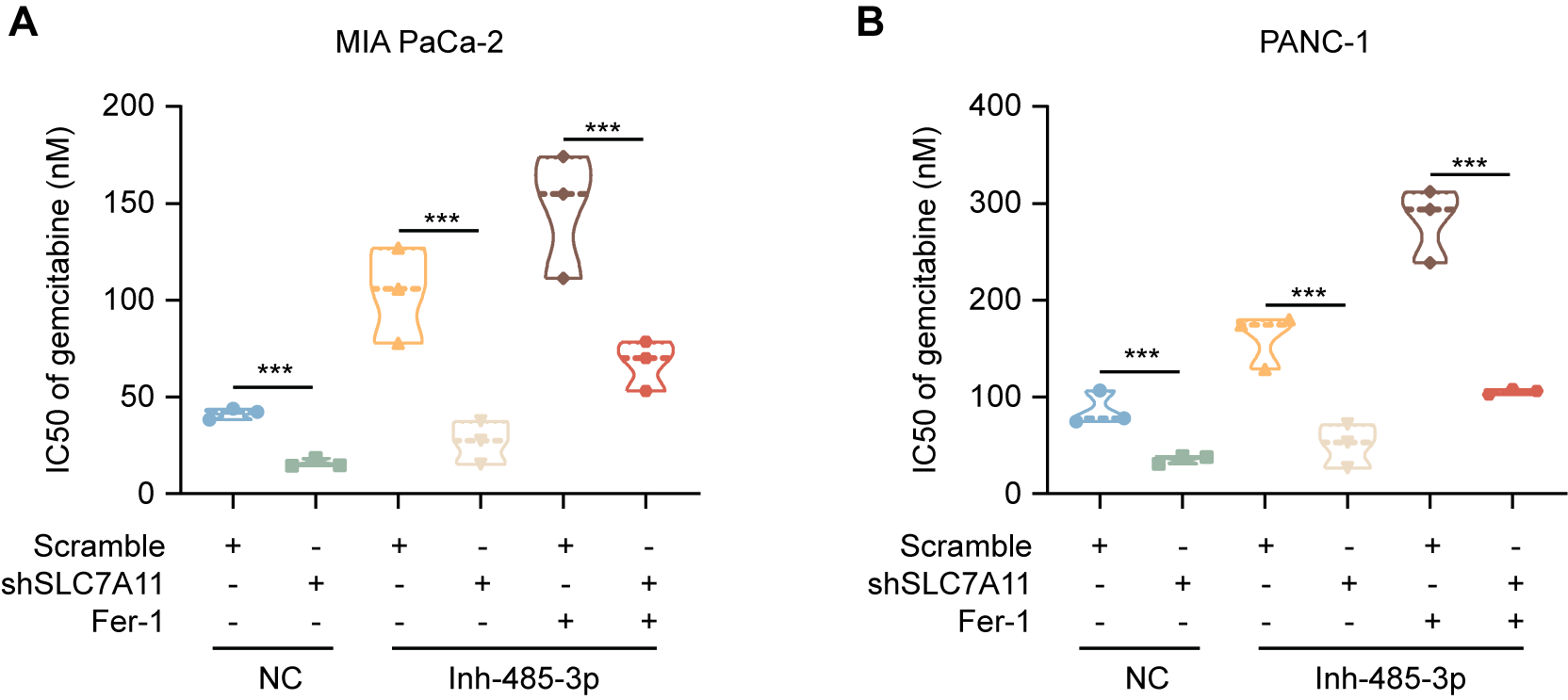

Supplement: Supplementary file 8 — Supplementary Figure S6 [file 41420_2024_2035_MOESM8_ESM.tif]

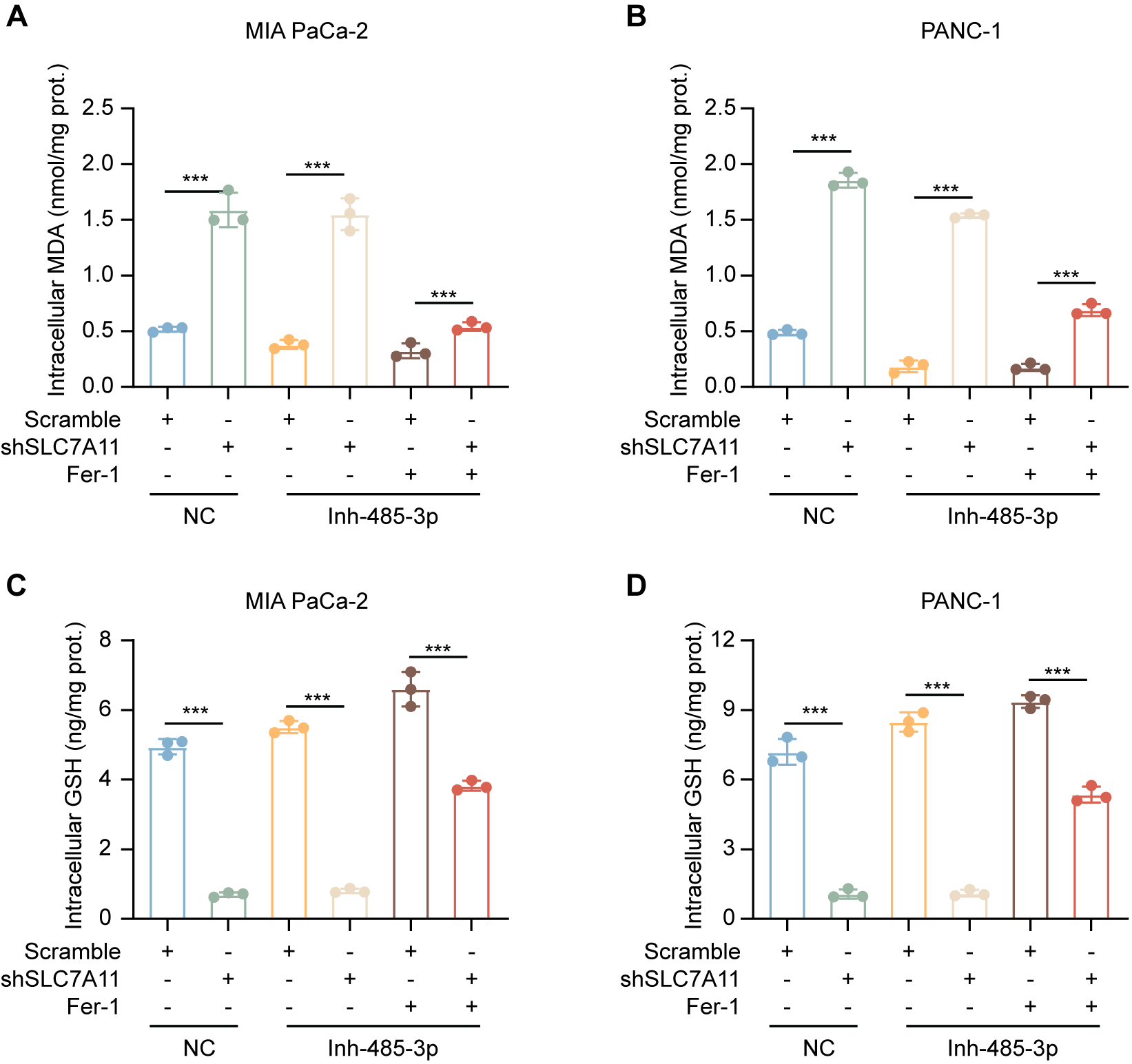

Supplement: Supplementary file 9 — Supplementary Figure S7 [file 41420_2024_2035_MOESM9_ESM.tif]

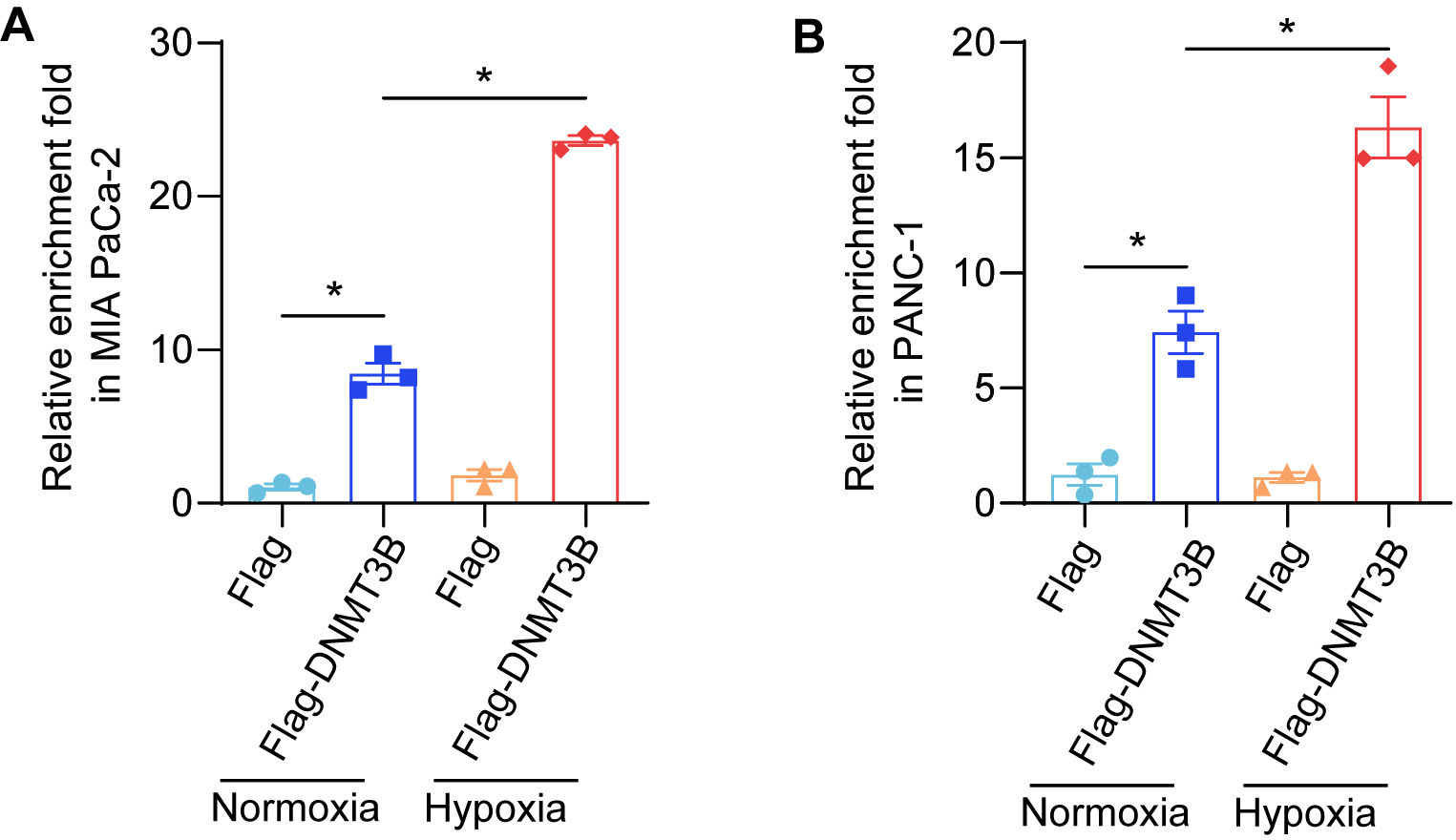

Supplement: Supplementary file 10 — Supplementary Figure S8 [file 41420_2024_2035_MOESM10_ESM.tif]

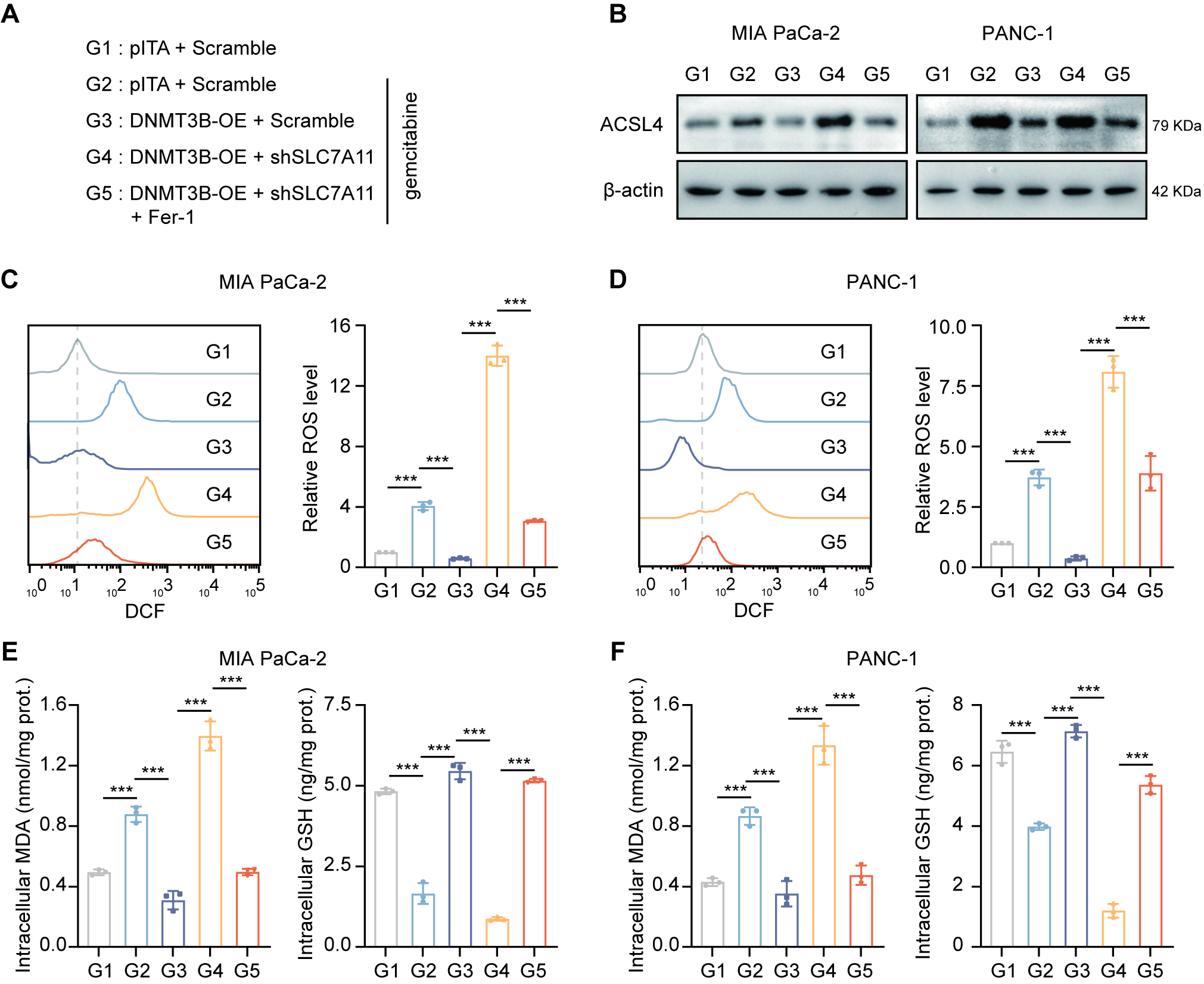

Supplement: Supplementary file 11 — Supplementary Figure S9 [file 41420_2024_2035_MOESM11_ESM.tif]
