# Supplementary material for: Hypoxia-induced epigenetic regulation of miR-485-3p promotes stemness and chemoresistance in pancreatic ductal adenocarcinoma via SLC7A11-mediated ferroptosis
Source: Cell Death Discov. 2024 May 29;10:262. doi: 10.1038/s41420-024-02035-x (PMC11137092; doi:10.1038/s41420-024-02035-x)
Supplement: Supplementary file 12 — Supplementary figure legends [file 41420_2024_2035_MOESM12_ESM.docx]

**Supplementary Figure Legends:**

**Supplementary Figure 1.** Western blot analysis of hypoxic marker HIF-1α in MIA PaCa-2 and PANC-1 cells, with the hypoxic condition in vitro imitated by physical- (A) or CoCl_2_(B) induced- method.

**Supplementary Figure 2.** MiR-485-3p inhibited the resistance of pancreatic cancer cells to gemcitabine. **A, C.** qPCR analysis of miR-485-3p expression in wild-type (WT), gemcitabine-resistant (GR) and miR-485-3p overexpressing pancreatic cancer cells MIA PaCa-2 (A) and PANC-1 (C). **B, D.** CCK-8 analysis of the sensitivity to gemcitabine of drug-resistance PDAC cells with miR-485-3p overexpression. Data are expressed as mean ± SD from three independent experiments. *P < 0.05; **P < 0.01; ***P < 0.001.

**Supplementary Figure 3.** Predicted targets of miR-485-3p in pancreatic cancer tissue. **A.** 49 candidate targets of miR-485-3p were shown (http://starbase.sysu.edu.cn). **B, C.** Correlation analysis between SOX9 (B), SLC7A11 (C) and miR-485-3p expression was performed using TCGA data, and the Pearson correlation coefficient was calculated. **D, E.** MiR-485-3p expression in pLV-485-3p (miR-485-3p overexpression) and Inh-485-3p (miR-485-3p silencing) pancreatic cancer cells, MIA PaCa-2 (D) and PANC-1 (E).

**Supplementary Figure 4. A.** MiR-485-3p expression in NC and Inh-485-3p (miR-485-3p silencing) pancreatic cancer cells (MIA PaCa-2 and PANC-1) with or without hypoxia. **B, C.** The expression levels of SOX9 and SLC7A11 in MIA PaCa-2 (B) and PANC-1 (C) with miR-485-3p silencing and/or hypoxia.

**Supplementary Figure 5.** Western blot analysis of stem cell markers SOX2 in PDAC cells MIA PaCa-2 (A) and PANC-1 (B) with miR-485-3p overexpression and exposed to normoxia or hypoxia.

**Supplementary Figure 6.** IC50 of gemcitabine in MIA PaCa-2 (A) and PANC-1 (B) cells exposed to hypoxia with knockdown of miR-485-3p and/or SLC7A11, combined with treatment with Fer-1.

**Supplementary Figure 7.** Intracellular MDA level of pancreatic cancer cells MIA PaCa-2 (**A**) and PANC-1 (**B**) cells exposed to hypoxia with knockdown of miR-485-3p and/or SLC7A11, combined with treatment with Fer-1. Intracellular GSH concentration of pancreatic cancer cells MIA PaCa-2 (**C**) and PANC-1 (**D**) cells exposed to hypoxia with knockdown of miR-485-3p and/or SLC7A11, combined with treatment with Fer-1.

**Supplementary Figure 8.** DNMT3B binding to the miR-485-3p promoter was determined by chromatin immunoprecipitation-RT-PCR in pancreatic cancer cells MIA PaCa-2 (**A**) and PANC-1 (**B**) under hypoxic conditions.

**Supplementary Figure 9.** DNMT3B-SLC7A11 axis regulates ferroptosis in PDAC cells treated with gemcitabine. **A.** Schematic illustration for the experimental design**. B.** The expression levels of ferroptosis indictor ACSL4 in MIA PaCa-2 and PANC-1 with DNMT3B overexpression and/or SLC7A11 knockdown combined with gemcitabine or Fer-1 treatment. **C, D.** Intracellular ROS levels of Mia PaCa-2 (C) and PANC-1 (D) cells with DNMT3B overexpression and/or SLC7A11 knockdown combined with gemcitabine or Fer-1 treatment. **E, F.** MDA and GSH levels in MIA PaCa-2 (E) and PANC-1(F) with DNMT3B overexpression and/or SLC7A11 knockdown combined with gemcitabine or Fer-1 treatment.
