# Supplementary material for: Hypoxia-induced epigenetic regulation of miR-485-3p promotes stemness and chemoresistance in pancreatic ductal adenocarcinoma via SLC7A11-mediated ferroptosis
Source: Cell Death Discov. 2024 May 29;10:262. doi: 10.1038/s41420-024-02035-x (PMC11137092; doi:10.1038/s41420-024-02035-x)
Supplement: Supplementary file 13 — Supplementary table legends [file 41420_2024_2035_MOESM13_ESM.docx]

**Supplementary Table Legends:**

**Supplementary Table S1.** The sequences of lentivirus or plasmids used in this study.

**Supplementary Table S2.** List of primers for qPCR used in this study.
