# Supplementary material for: Hypoxia-induced epigenetic regulation of miR-485-3p promotes stemness and chemoresistance in pancreatic ductal adenocarcinoma via SLC7A11-mediated ferroptosis
Source: Cell Death Discov. 2024 May 29;10:262. doi: 10.1038/s41420-024-02035-x (PMC11137092; doi:10.1038/s41420-024-02035-x)

Fig. 2G

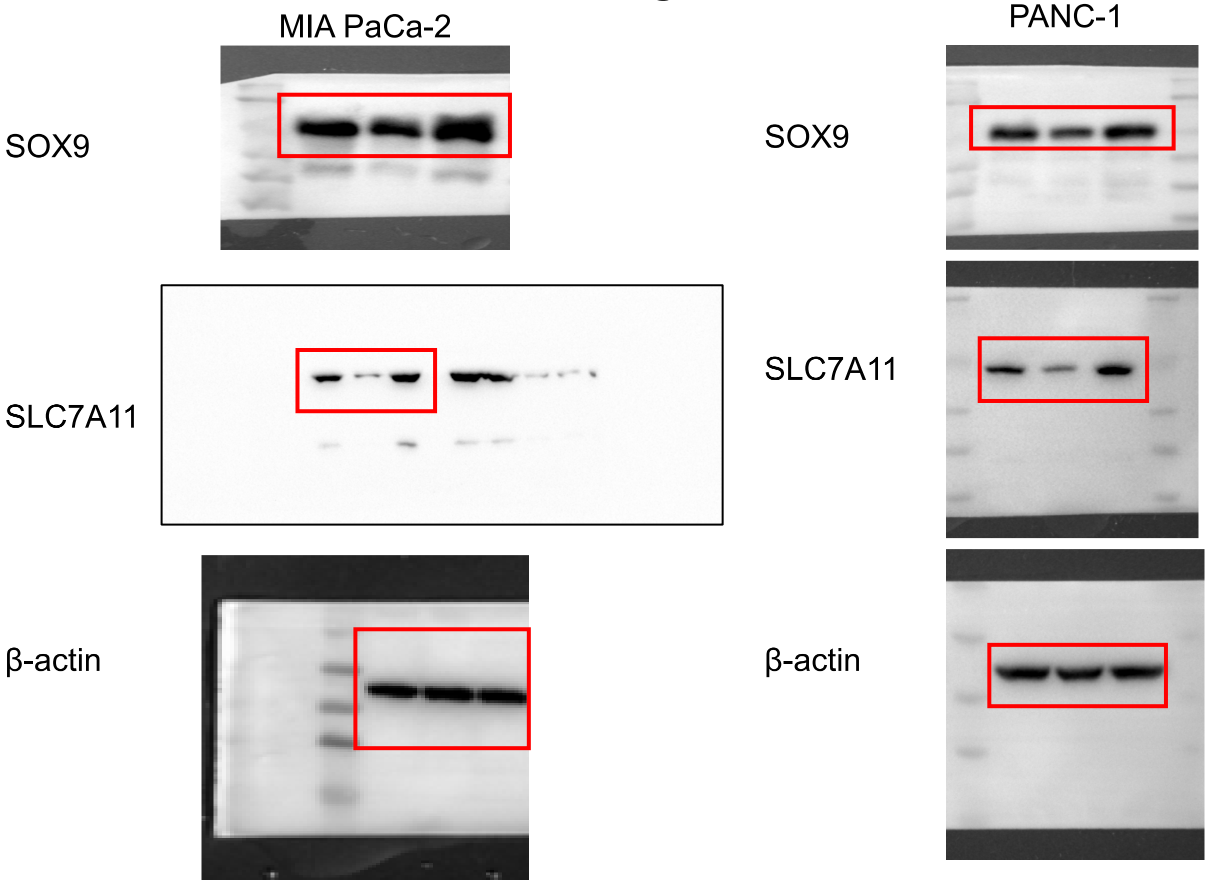

Fig.2H

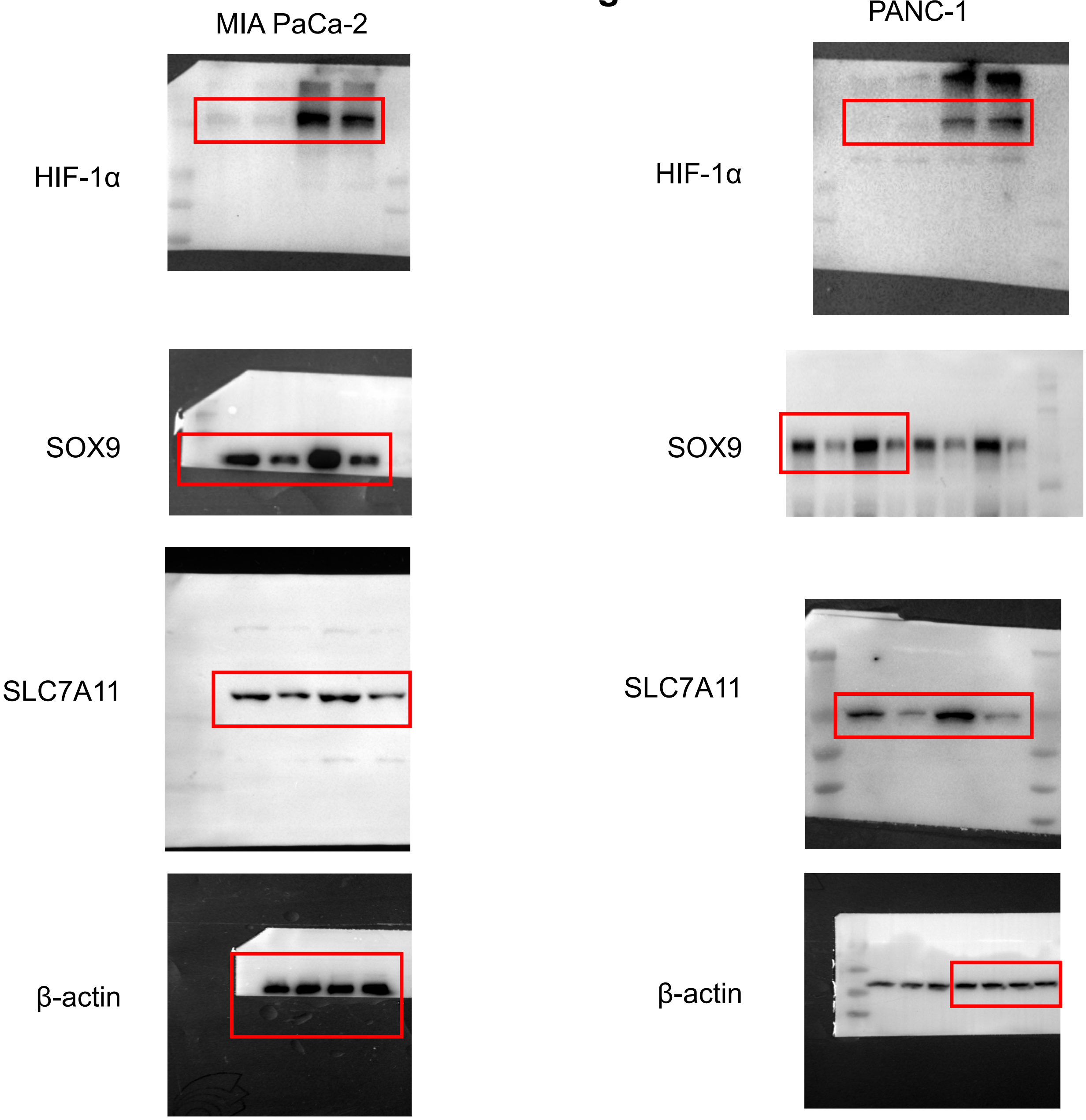
MIA PaCa-2
PANC-1
HIF-1α
HIF-1α
SOX9
SOX9
SLC7A11
SLC7A11
β-actin
β-actin

Fig.5A

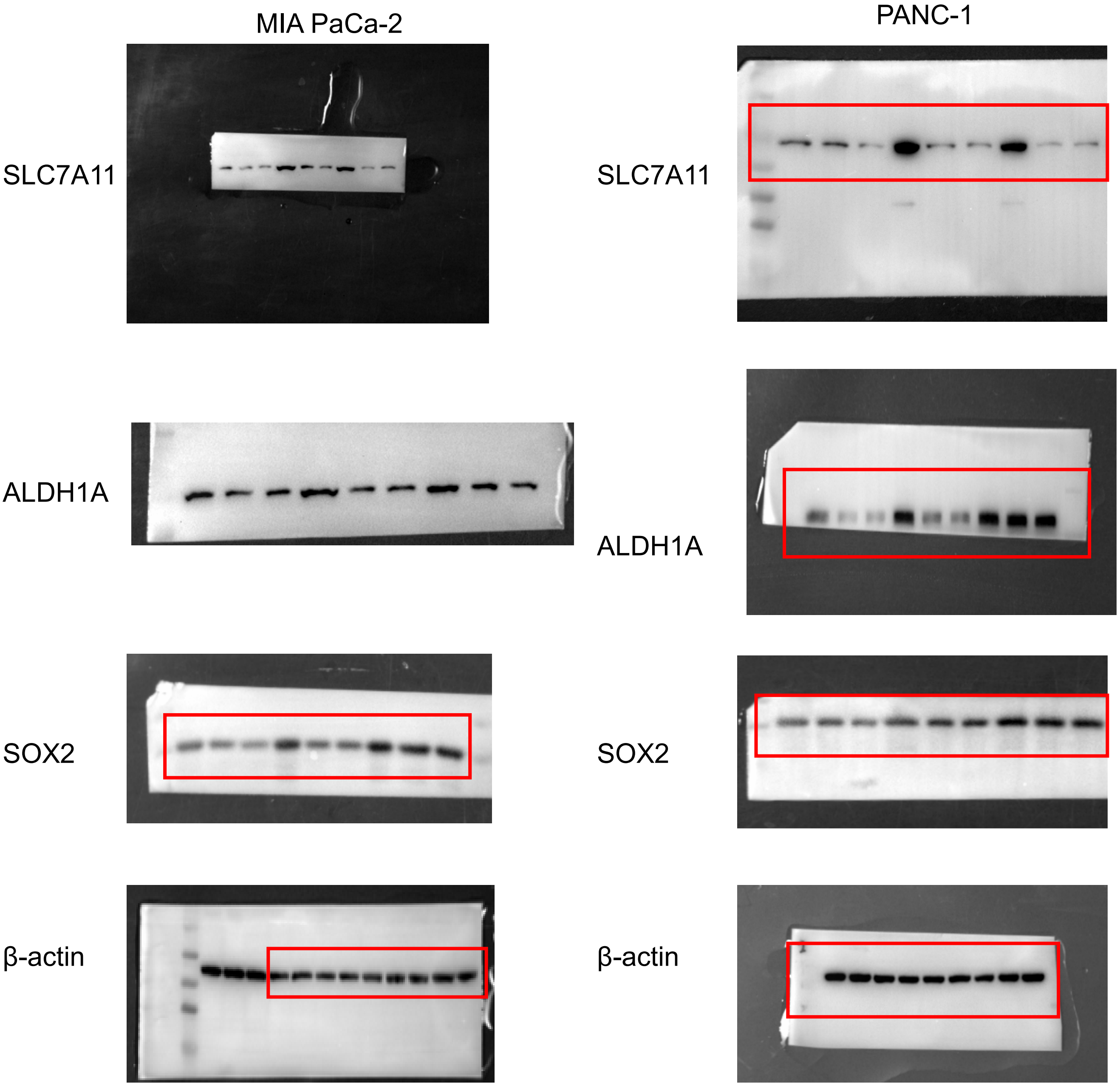
MIA PaCa-2
PANC-1
SLC7A11
SLC7A11
ALDH1A
ALDH1A
SOX2
SOX2
β-actin
β-actin

Fig.6A

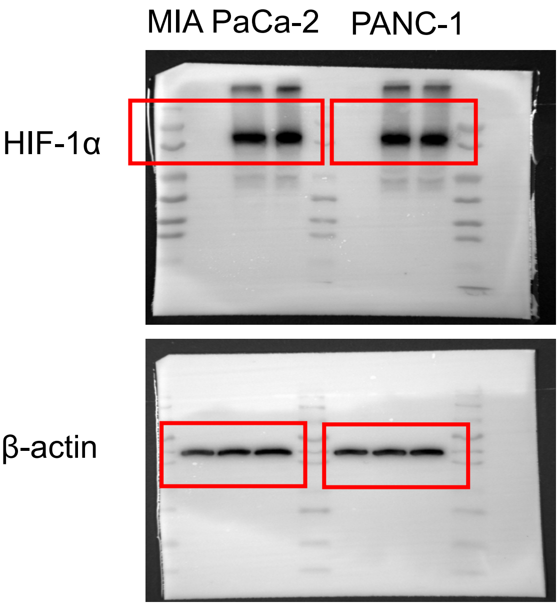

Fig.6B

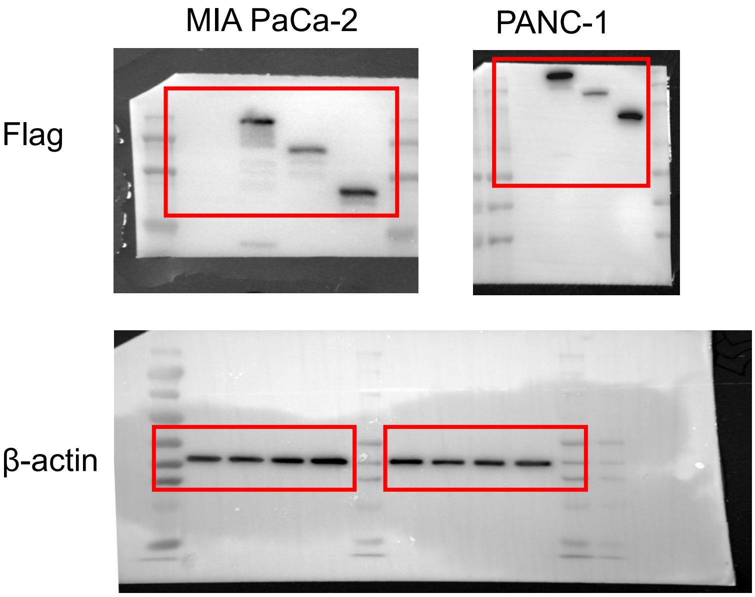

Fig.6C

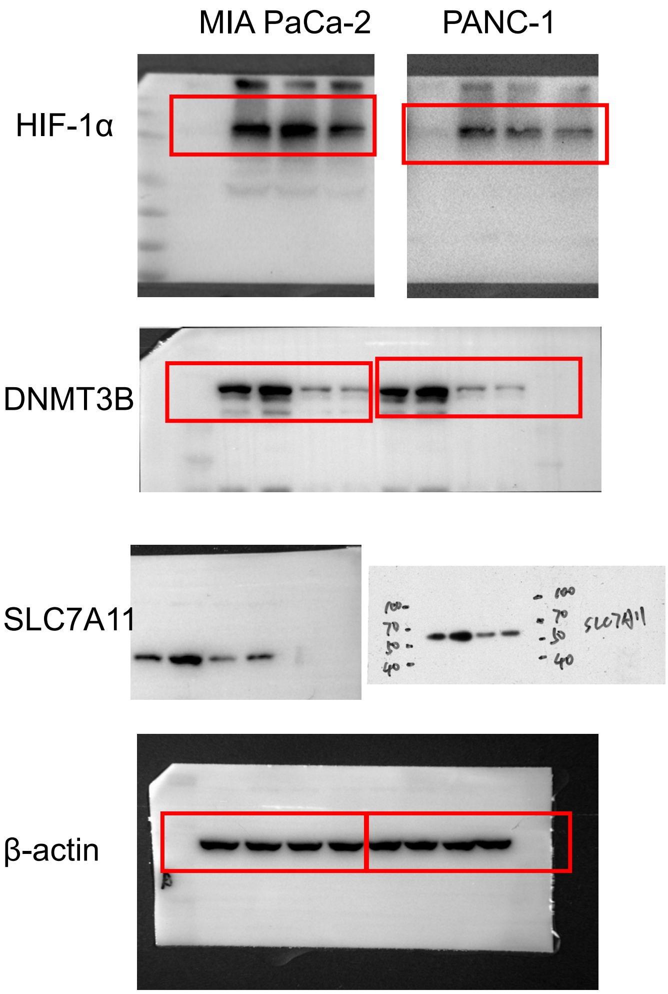

Fig.6D

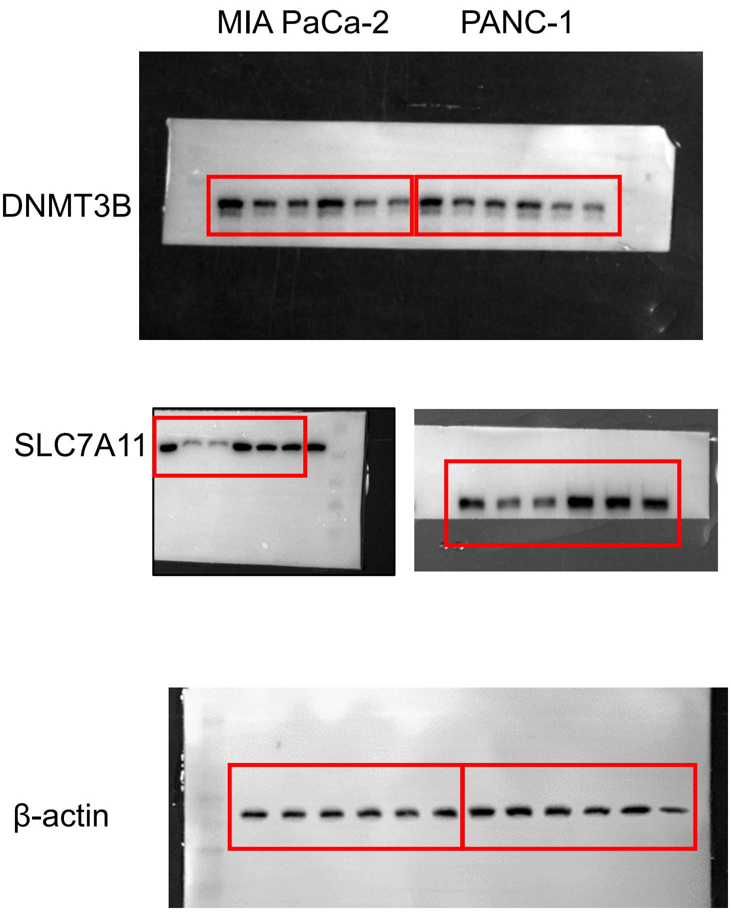

Fig.7B

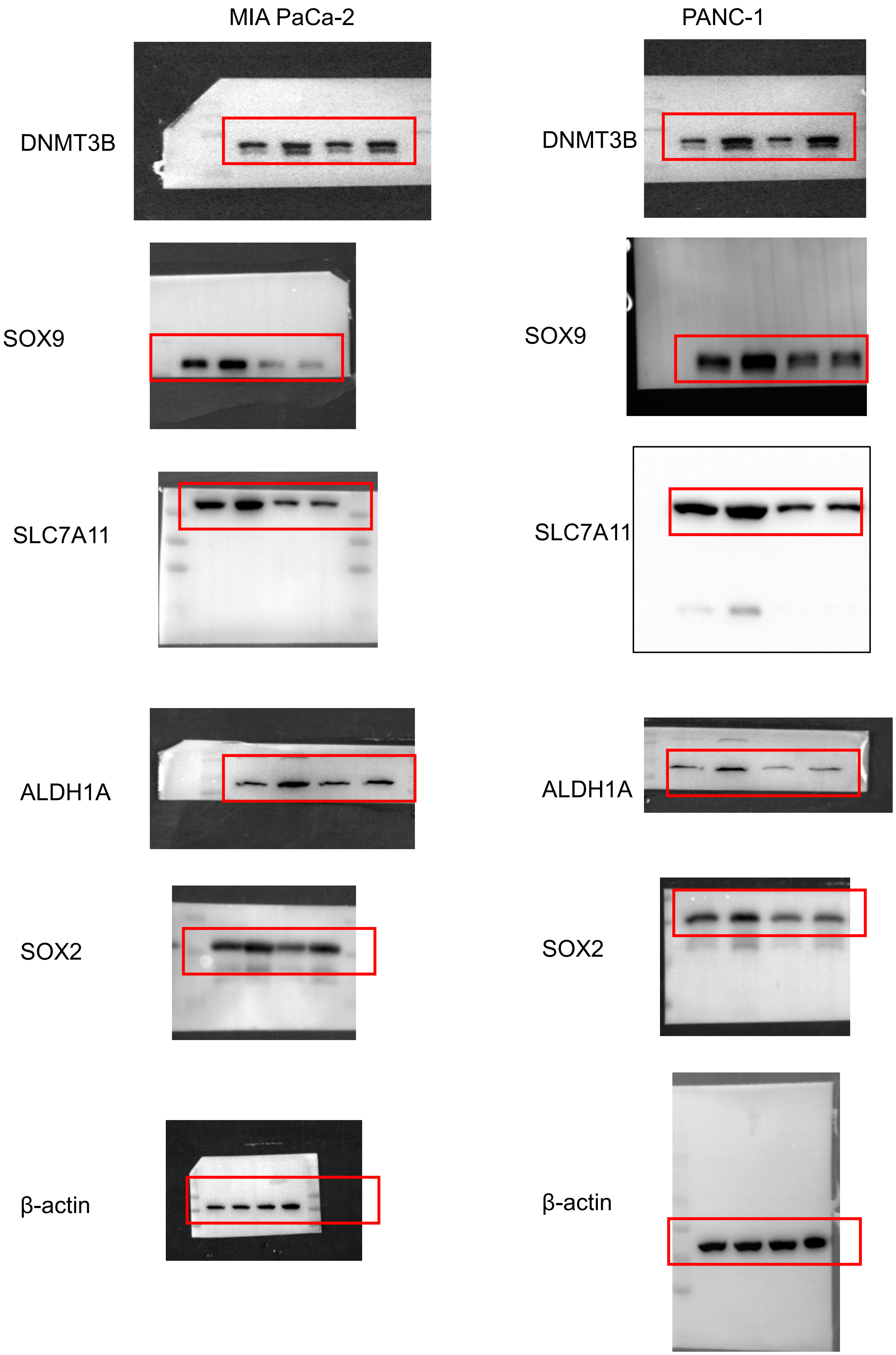
MIA PaCa-2
PANC-1
DNMT3B
SOX9
SLC7A11
ALDH1A
SOX2
β-actin
DNMT3B
SOX9
SLC7A11
ALDH1A
SOX2
β-actin

Supplementary Fig. 1A

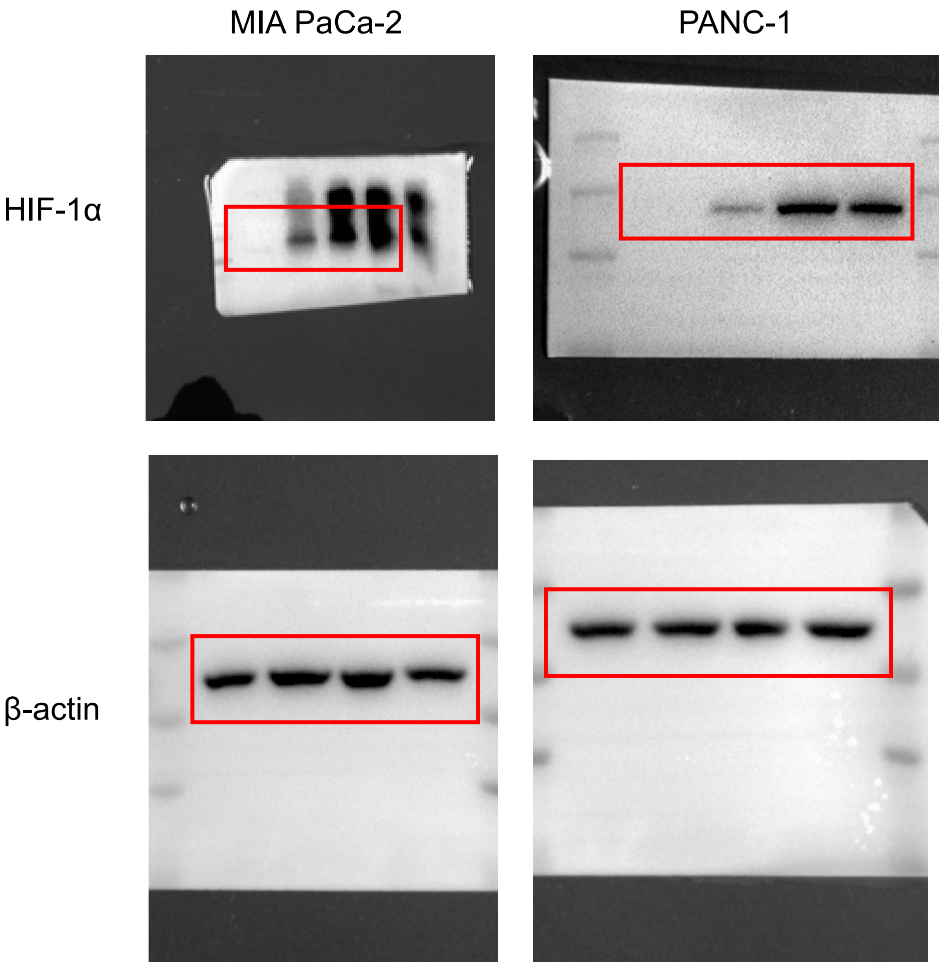

Supplementary Fig. 1B

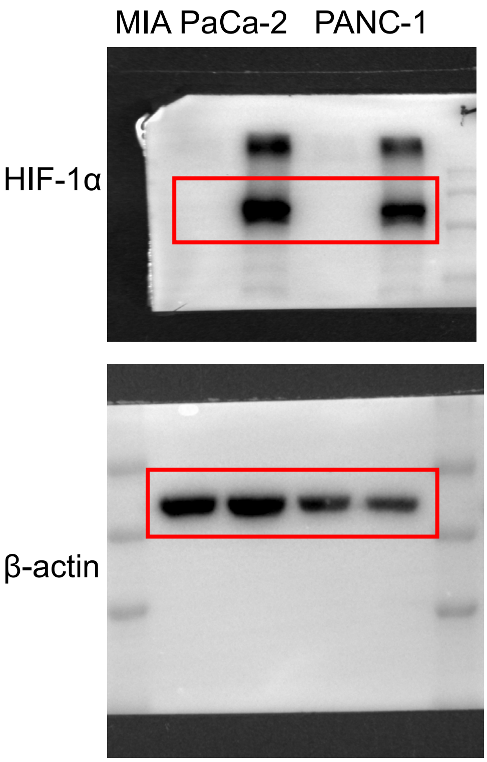

Supplementary Fig. 4A Supplementary Fig. 4B

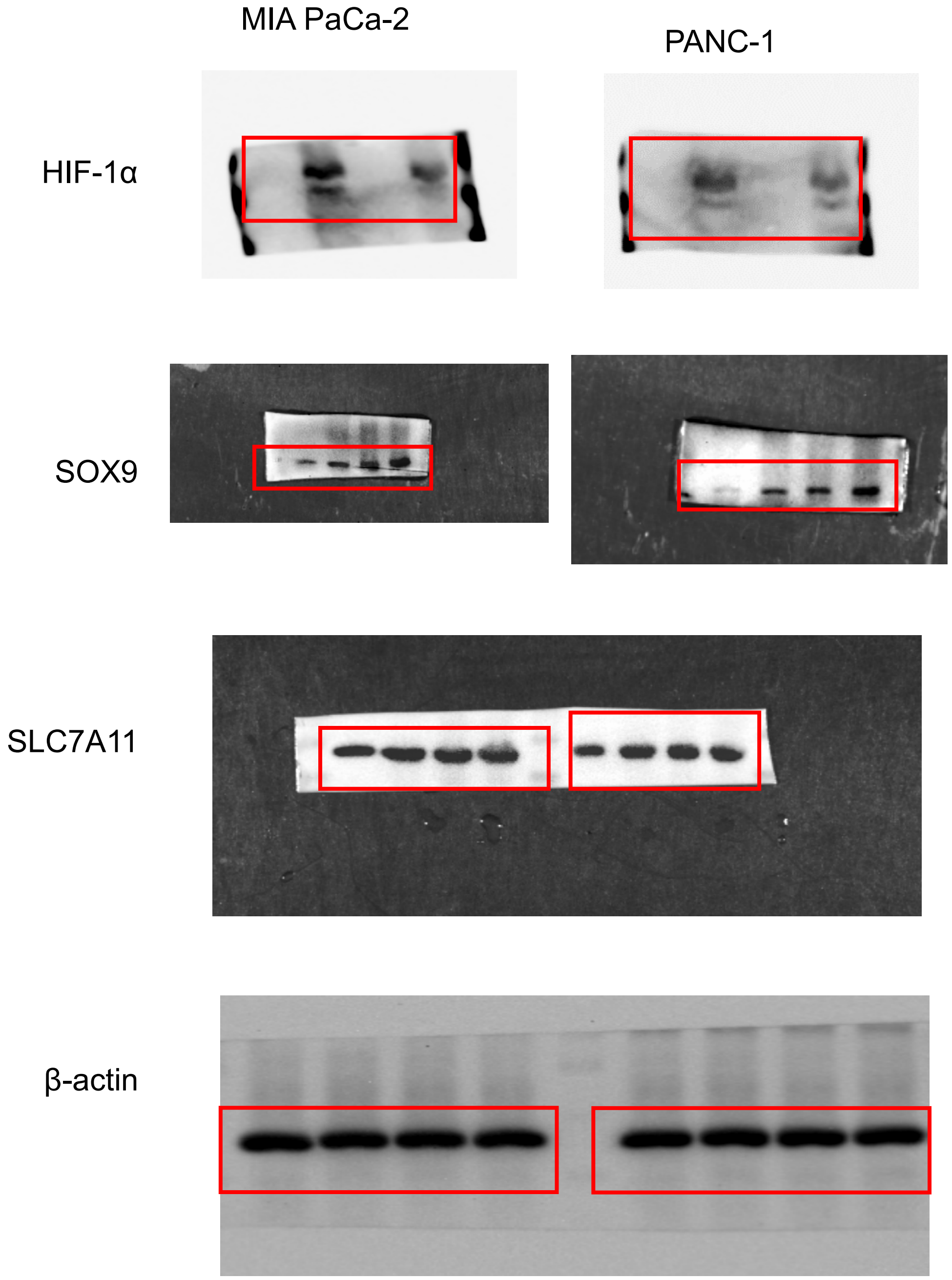

Supplementary Fig. 5A

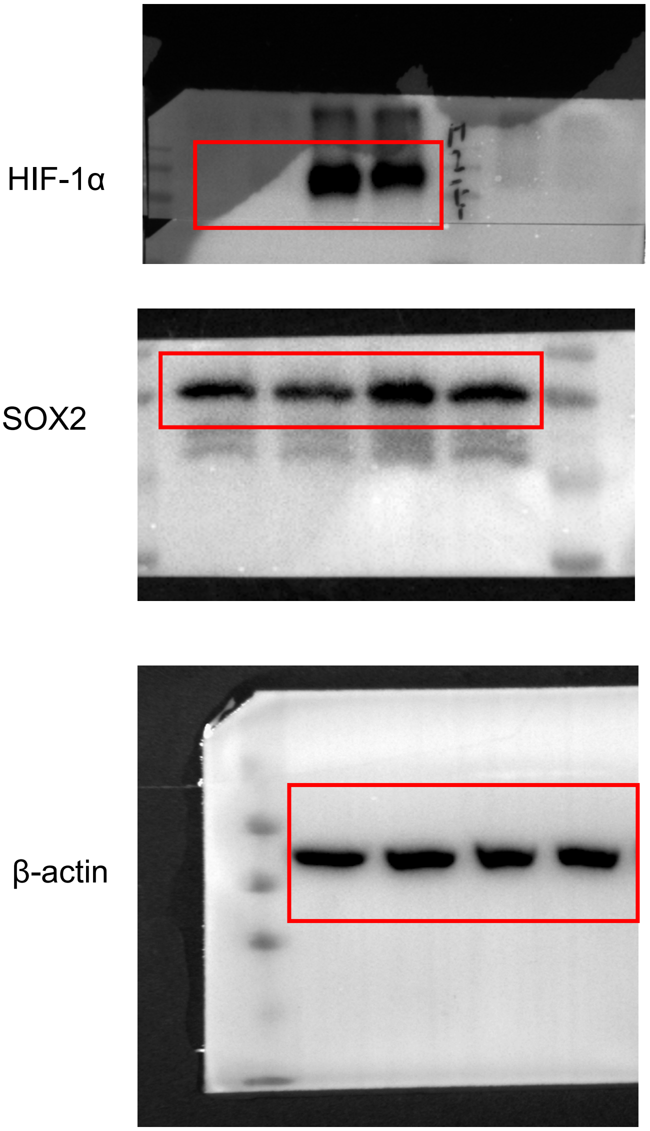

Supplementary Fig. 5B

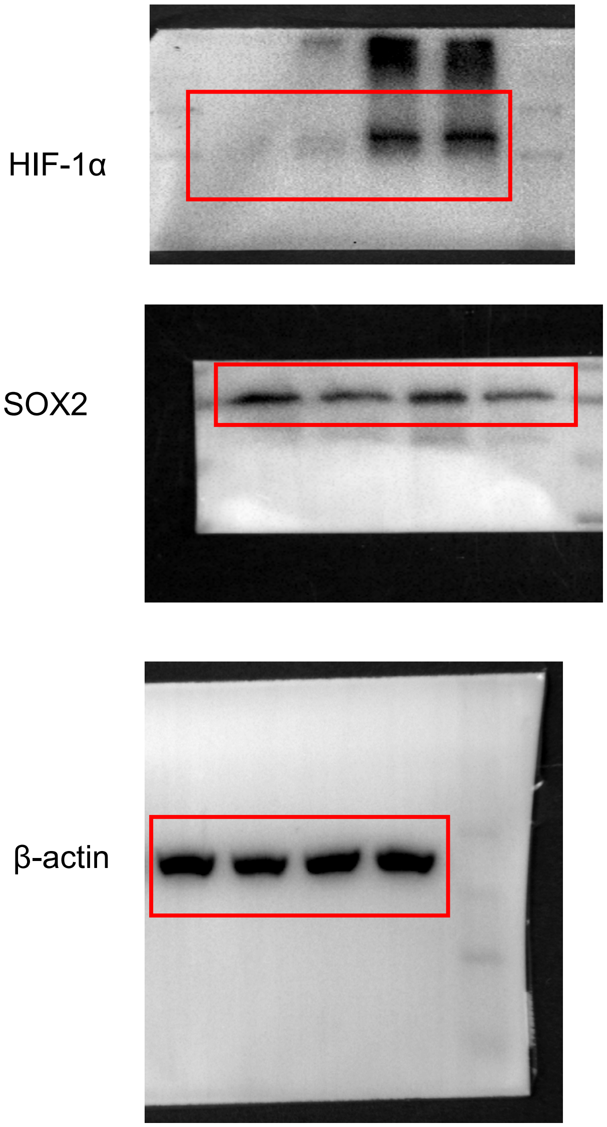

Supplementary Fig. 9B

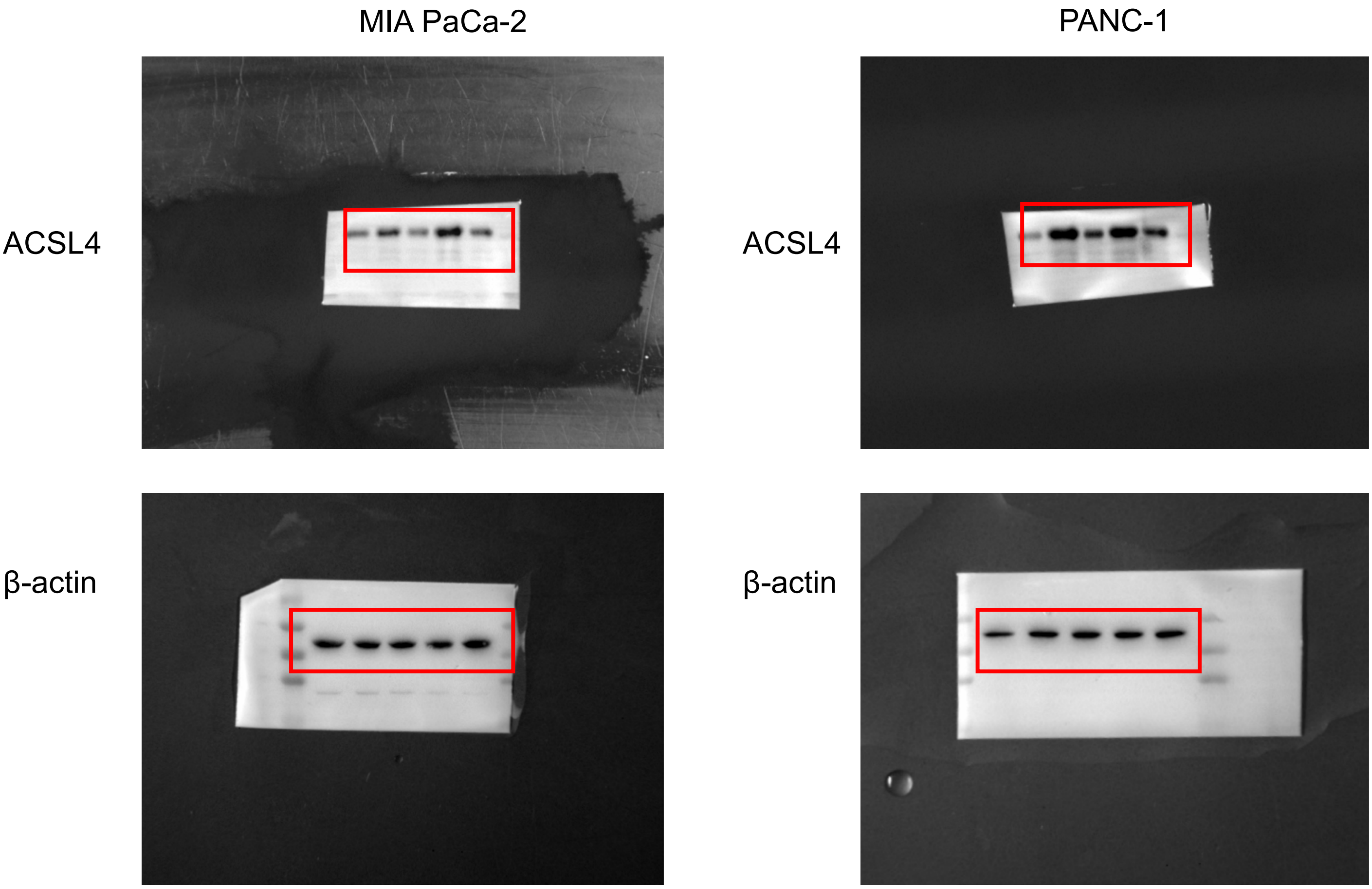

Supplement: Supplementary file 14 — Full and uncropped western blots [file 41420_2024_2035_MOESM14_ESM.pdf]
